# Paleo-evolutionary plasticity of plant disease resistance genes

**DOI:** 10.1186/1471-2164-15-187

**Published:** 2014-03-12

**Authors:** Rongzhi Zhang, Florent Murat, Caroline Pont, Thierry Langin, Jerome Salse

**Affiliations:** 1 INRA/UBP UMR 1095 GDEC ‘Génétique, Diversité et Ecophysiologie des Céréales’, 5 chemin de Beaulieu, 63100 Clermont-Ferrand, France; 2 Institute of Crop Sciences, Shandong Academy of Agricultural Sciences, Jinan 250100, China

**Keywords:** R-genes, Duplication, Plasticity, Evolution

## Abstract

**Background:**

The recent access to a large set of genome sequences, combined with a robust evolutionary scenario of modern monocot (*i.e.* grasses) and eudicot (*i.e.* rosids) species from their founder ancestors, offered the opportunity to gain insights into disease resistance genes (R-genes) evolutionary plasticity.

**Results:**

We unravel in the current article (*i*) a R-genes repertoire consisting in 7883 for monocots and 15758 for eudicots, (*ii*) a contrasted R-genes conservation with 23.8% for monocots and 6.6% for dicots, (*iii*) a minimal ancestral founder pool of 384 R-genes for the monocots and 150 R-genes for the eudicots, (*iv*) a general pattern of organization in clusters accounting for more than 60% of mapped R-genes, (*v*) a biased deletion of ancestral duplicated R-genes between paralogous blocks possibly compensated by clusterization, (*vi*) a bias in R-genes clusterization where Leucine-Rich Repeats act as a ‘glue’ for domain association, (*vii*) a R-genes/miRNAs interome enriched toward duplicated R-genes.

**Conclusions:**

Together, our data may suggest that R-genes family plasticity operated during plant evolution (*i*) at the structural level through massive duplicates loss counterbalanced by massive clusterization following polyploidization; as well as at (*ii*) the regulation level through microRNA/R-gene interactions acting as a possible source of functional diploidization of structurally retained R-genes duplicates. Such evolutionary shuffling events leaded to CNVs (*i.e.* Copy Number Variation) and PAVs (*i.e.* Presence Absence Variation) between related species operating in the decay of R-genes colinearity between plant species.

## Background

Pathogen attacks from fungi [1], viruses [2], nematodes [3] or bacteria [4], compelled plants to prevent damages by engaging an “arms race” with these organisms. Therefore, plants have developed a battery of defense mechanisms involving (1) PTI (PAMP*-*Triggered Immunity) triggered by PAMP (Pathogen-Associated Molecular Patterns) [5-7] and (2) ETI (Effector*-*Triggered Immunity) triggered by effectors leading to hypersensitive response (referenced as HR [8]). Therefore, constant evolution leading to novel mechanisms is crucial for plant defense processes as well as adaptation to biotic stresses. The most studied disease resistance proteins encoding genes (hereafter R-genes) or genes involved in disease resistance pathways are Nucleotide-Binding-Sites (NBS) [9,10], Leucine-Rich Repeats (LRR) [9,10], Toll-Interleukine1 Receptors (TIR), WRKY transcription factors [11,12], Lysine Motif (LysM) families [13,14], and Protein Kinase families (hereafter referenced as PKinase) [15,16]. R-genes can then be functionally classified into five distinct groups consisting in CNL (genes encoding proteins with coiled-coil, nucleotide binding site, leucine-rich repeat domains, *i.e.* CC-NBS-LRR), TNL (genes encoding proteins with Toll-interleukin receptor-like, nucleotide binding site, leucine-rich repeat domains, *i.e.* TIR-NBS-LRR), RLP (genes encoding proteins with receptor serine-threonine kinase like, extracellular leucine rich repeat domains, *i.e.* ser/thr-LRR), RLK (genes encoding proteins with kinase, extracellular leucine-rich repeat domains, *i.e.* Kin-LRR), and RGA (includes all other genes conferring resistance through different molecular mechanisms) classes [17]. LRR-RLK, LRR, LysM, LysM-kinase act as pattern-recognition receptors (PRR) involved in the PTI pathway, while NBS-LRR commonly responds in the frame of the ETI pathway [18,19]. Finally, WRKY and protein-kinases, associated with protein domains encoded by R-genes (hereafter R-domains), can also be activated by PRRs in disease resistance pathways [18-20].

R-genes have been reported to be ancient and conserved genes that have been detected in gymnosperms, plants and animals to ensure immunity [21-23]. However, comparative genomic analyses have shown that R-genes are associated with a great structural diversity in vertebrates and plants. For example, the presence of TIR domains in conifers and mosses indicated that TIR may represent an ancestral R-gene family with shared functionality with their mammalian or insect homologues regarding innate immunity [21,22,24-26]. TIR genes typically expanded in eudicot genomes, while they have been reported to be absent (or at least rare) in grass genomes [27-31]. Moreover, tandem and segmental duplications have been reported as a source of structural plasticity of NBS-LRR genes in plant genomes [32]. Furthermore, PAV (Presence/Absence Variation) polymorphisms often exist in a population or between species [33-36]. Overall, small-scale studies (*i.e.* few R-genes families/domains and/or few plant species investigated) have suggested R-genes as one of the most plastic gene family in plants associated with intense structural shuffling in the course of evolution leading to synteny erosion or alternatively loss [37]. For example, evolutionary investigations of R-genes in *Arabidopsis* and rice have been conducted suggesting contrasted amplification of TNL and CNL families as well as clusterization of NBS-LRRs *via* segmental and tandem duplications or ectopic gene conversions [38].

Few studies have investigated the conservation of R-genes across a large set of plant species and at the whole-genome level. Genome sequences from flowering plants that are derived from a common ancestor 135 to 250 million years ago (mya) are increasingly available in the public domain for evolutionary studies. Recent paleohistorical studies demonstrated that modern grass genomes, including Panicoideae (sorghum [*Sorghum bicolor*], [39] maize [*Zea mays*], [40]), Ehrhartoideae (rice [*Oryza sativa*], [41]), and Pooideae (*Brachypodium distachyon*; [42]), were shaped from n = 5 to 12 ancestral grass karyotypes (AGKs) containing a minimal set of 6045 ordered protogenes with a minimum physical size of 33 Mb [43-45] through whole-genome duplication (WGD) and ancestral chromosome fusion events. Likewise, the recent comparison of numerous eudicot genomes (*i.e.* mainly eurosids), including grape (*Vitis vinifera*; [46]), poplar [47], *Arabidopsis thaliana*[48], soybean *Glycine max*; [49], and cacao (*Theobroma cacao*; [50]), revealed that modern eudicot genomes derived from an n = 7 ancestor that went through a paleohexaploidization event to reach a n = 21 intermediate followed by numerous lineage-specific WGDs and chromosome fusion events [46,51]. During the last 135 to 250 million years of evolution, the protein-coding gene families have been then shaped by various gene duplication mechanisms, including WGDs (or polyploidization), segmental duplications, and tandem duplications. It is now well established that all modern diploid plant species are highly shuffled paleopolyploids [52-55].

Duplication (WGDs, segmental duplications and tandem duplications) were proposed as the major mechanisms driving R-genes family expansion or contraction from their traceable ancestral copies [56-58]. However, a systematic and detailed study of the paleohistorical evolution of R-genes across plant subfamilies including rosids species (*Arabidopsis thaliana*[48], Grape [59], Apple [60], Poplar [47], Soybean [49], Lotus [61], Strawberry [62], Cacao [50] and Papaya [63]), and grasses (Rice [41], Maize [40], *Sorghum bicolor*[39] and *Brachypodium distachyon*[42]) is still lacking. Particularly, how R-genes have behaved following polyploidization events is not well established. Such a precise investigation of the paleohistory of R-genes during the last 250 million years of evolution will unravel precise mechanisms that lead to the reduced conservation of R-genes observed between modern plant species.

## Results

### Disease resistance gene mapping, conservation and evolutionary patterns

To identify the largest set of plant R-genes, three complementary methods (see Methods section) were combined (illustrated as Additional file 1: Figure S1) consisting in (1) the detection of PFAM [64] domains, (2) the exploitation of public genome annotations, and (3) the use of the Plant Resistance Gene Database, PRGdb (http://prgdb.cbm.fvg.it/index.php, [65]). The integration of the three previous approaches allowed to construct a non-redundant set of putative R-genes in the plant species considered in this study (Table 1, Additional file 1: Figure S2 and Additional file 2: Dataset S1). Based on the genome annotation approach, 2697 R-genes were identified in monocots, and 3021 in eudicots, corresponding to a total set of 5718 plant R-genes (Table 1). Regarding the PFAM domain identification procedure, 8013 sequences in monocots and 14996 in eudicot species were identified, corresponding to 23009 putative R-genes sequences in total (Table 1). Finally, the PRGdb aligned on the 13 genome sequences investigated unraveled 1874 sequences in monocots and 2001 sequences in eudicot species. These three complementary methods lead us to deliver the most complete and non-redundant list in angiosperms consisting in 23641 R-genes sequences, 7883 for the monocots and 15758 for the eudicots (*cf* Table 1 and Additional file 2: Dataset S1). The identified R-genes families deriving from the annotation, PFAM and PRGdb approaches are detailed in Table 1 and Additional file 1: Figure S2, showing that the total number of non-redundant R-genes in plants is close to the dataset obtained with the PFAM detection approach, suggesting that such method is the most appropriate in delivering the largest and most complete set of R-genes in any genomic sequence of interest.

**Table 1:** R-genes catalog and conservation in plant genomes

**Species**	**Genome data**				**R-genes data set**				**R-genes conservation**	
	**Nb chr.**	**size (Mbp)**	**Nb gene**	**Nb ortholog**	**Annot**	**Pfam**	**PRG db**	Non-redundant R-genes*	**OrthoSeq**	**%**
**Monocot**										
*Oryza sativa (rice)*	12	372	41046	*Ref*	1180	2294	1316	**2637**(889;539;10;16;100;1544;214;102)	*Ref*	*Ref*
*Sorghum bicolor*	10	659	34008	6147(14,9%)	570	1616	159	**1717**(516;284;2;20;98;1158;68;39)	**413**(116;16;0;7;5;347;19;3)	24,1%
*Zea mays (maize)*	10	2365	32540	4454(10,85%)	480	2630	399	**1867**(558;140;13;15;106;1255;0;45)	**319**(104;6;2;3;3;261;0;6)	17,1%
*Brachypodium distachyon*	5	271	25504	8533(20,78%)	467	1473	nd	**1662**(435;199;2;11;80;1094;0;104)	**495**(137;19;0;4;7;417;0;1)	29,8%
**Monocot Total**	**-**	**-**	**133098**	**-**	**2697**	**8013**	**1874**	**7883**(2398;1162;27;62;384;5051;282;290)	**1227**(357;41;2;14;15;1025;19;10)	**-**
**Eudicot**										
*Vitis vinifera (grape)*	19	302	21189	*Ref*	256	892	170	**1078**(421;229;50;10;37;557;0;91)	*Ref*	*Ref*
*Arabidopsis thaliana*	5	119	33198	2389(11,27%)	564	1407	1105	**1559**(436;168;125;12;73;1010;3;126)	**74**(27;0;0;1;0;58;0;2)	4,7%
*Populus trichocarpa (poplar)*	19	294	30260	4555(21,5%)	212	1229	134	**1297**(413;122;31;24;63;930;0;32)	**122**(53;4;1;4;6;89;0;2)	9,4%
*Carica papaya*	9	234	19205	3199(15,1%)	113	674	nd	**703**(200;50;13;11;42;515;0;0)	**101**(45;1;1;1;4;74;0;0)	14,4%
*Glycine max (soybean)*	20	949	46164	4013(18,94%)	1023	3104	318	**3310**(1097;411;166;49;179;2172;170;146)	**148**(51;0;1;3;8;114;6;3)	4,5%
*Malus x domestica (apple)*	17	742	58979	3498(16,51%)	853	4135	243	**4252**(1638;860;340;9;123;2292;0;117)	**125**(41;0;1;1;7;94;0;2)	2,9%
*Lotus japonicus*	6	500	15470	1720(8,12%)	*nd*	664	26	**668**(165;77;49;0;34;443;0;5)	**46**(18;1;0;0;2;36;0;1)	6,9%
*Fragaria vesca (strawberry)*	7	240	34809	3289(15,52%)	*nd*	1452	1	**1452**(483;154;148;3;52;884;0;0)	**108**(44;2;1;0;1;89;0;0)	7,4%
*Theobroma cacao*	10	430	27814	4472(20,1%)	*nd*	1439	4	**1439**(492;220;14;4;55;934;0;0)	**149**(55;3;0;1;10;113;0;0)	10,4%
**Eudicot Total**	**-**	**-**	**265899**	**-**	**3021**	**14996**	**2001**	**15758**(5345;2291;936;122;658;9737;173;517)	**873**(334;11;5;11;38;667;6;10)	**-**

*Number of non-redundant R-genes.

Nb, number.

Nd, not detected.

Numbers in bracket refers to LRR, NBS, TIR, LysM, WRKY, Pkinase, Ser/Thr and Disease resistance related genes respectively.

In bold, total non-redundant detected R-genes.

Ref, reference.

**Figure 1:**
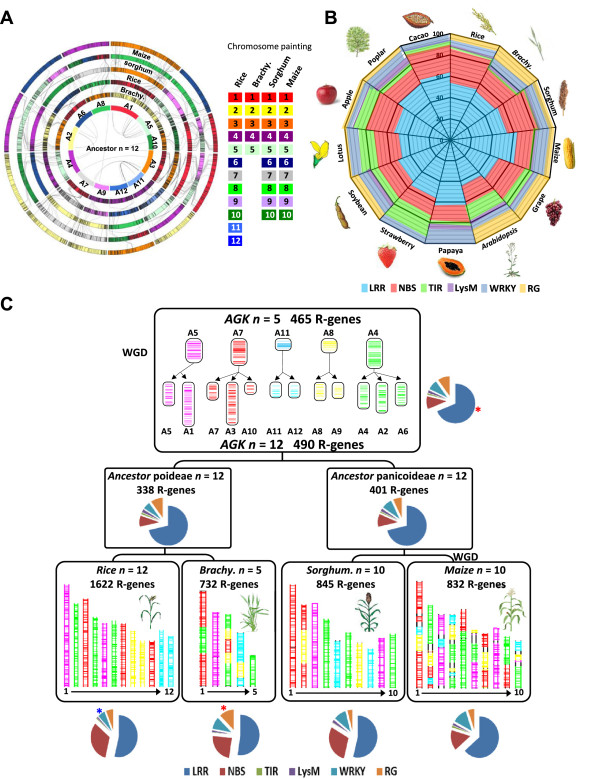
**R-genes conservation and evolution in plants. (A)** Grass genome synteny is illustrated as concentric circles. The chromosomes are highlighted with a color code (right) that illuminates the n = 12 monocot ancestral genome structure (inner circle A1 to A12). Any radius of the circle shows orthologous chromosomes between *Brachypodium*, rice, sorghum, and maize genomes. Maize genome is depicted as a double circle originating from the maize-specific recent WGD. Colinear R-genes are linked with black lines between circles, and ancestral duplicated R-genes are linked with black lines at the center of the circle. **(B)** R-genes content from 13 plant genomes including monocots (rice, *Brachypodium*, sorghum, and maize) and eudicots (*Arabidopsis*, Grape, Cacao, Papaya, Strawberry, Poplar, Lotus, Apple, and Soybean). The color code (bottom) highlights the R-gene classes investigated (LRR, NBS, TIR, LysM, RG). **(C)** Evolutionary scenario of R-genes in monocots. The modern grass genome structures (bottom) are depicted with a five-color code that illuminates their relationship with the n = 5 (A5, A7, A11, A8, A4) and n = 12 (A1 to A12) ancestors (top), according to Murat et al. [67]. The characterized R-genes are illustrated as vertical bars on the chromosomes of modern and ancestral genomes. The percentages of R-gene classes (LRR, NBS, TIR, LysM, RG, highlighted with the color code legend at the bottom) are shown with circular distributions for the four monocot genomes (bottom), the rice/*Brachypodium* and sorghum/maize ancestral genome intermediates (center), as well as for the ancestral karyotype (top). Statistically enriched and impoverished R-gene families are illustrated respectively with red and blue dots on the circular distributions.

The conservation of R-genes between genomes was then investigated using rice and grape as reference genomes respectively for monocots and eudicots as they represent the most closely related modern genome structures of the reconstructed plant ancestral karyotypes [66]. The 2637 rice R-gene sequences were aligned against the three other monocot species available, *i.e.* sorghum, *Brachypodium* and maize. Similarly, the 1078 grape R-genes sequences were aligned against the eight eudicot species investigated (Table 1, ‘Evolutionary Data’ column). 413 orthologous R-genes were identified in sorghum corresponding to 24.1% of the reference dataset (*i.e.* rice). Similarly, 319 orthologous R-genes were found in maize (17.1% of the reference) and 495 in *Brachypodium* (29.8% of the reference). This result suggests that R-genes may appear as more conserved than the total protein-coding genes, with respectively 24.4%, 17.1%, and 29.8% of rice orthologous R-genes characterized in sorghum, maize and *Brachypodium* compared to 14.9%, 10.85%, and 20.78% of overall annotated protein-coding genes conservation observed for the same species (*P-value* < 5% Fisher Exact Test, Additional file 1: Table S1). However, if the protein kinase family is excluded, as it consists in the largest (14788 kinases) one compared to the six others (with an average of 1264 R-genes for these families), the synteny conservation observed for R-genes is similar to one observed for the total annotated protein-coding genes. The R-genes synteny (excluding protein kinases) at the chromosome level between grasses is illustrated as Figure 1A. In contrast to grasses, in eudicot species R-genes are significantly less conserved than the annotated protein-coding genes (except for papaya) with 4.7%, 9.4%, 4.5%, 2.9%, 6.9%, 7.4%, and 10.4% orthologous R-genes identified in *Arabidopsis*, poplar, soybean, apple, lotus, strawberry, and cacao compared to 11.27%, 21.5%, 18.94%, 16.51%, 8.12%, 15.52%, and 20.10% for the conservation of total protein-coding genes for the same species (*P-value* < 5% Fisher’s Exact Test; Additional file 1: Table S1). This result may suggest a reduced syntenic conservation of R-genes in eudicots compared to monocots.

R-genes were classified into seven distinct groups, according to their specific encoded protein domains (R-domains). In monocots, we identified 2398 LRR, 1162 NBS, 27 TIR, 62 LysM, 384 WRKY, 5333 Protein-kinases, and 290 RG. In eudicots, 5345 LRR, 2291 NBS, 936 TIR, 122 LysM, 658 WRKY, 9910 Protein-kinases, and 517 RG were characterized. The distribution of the R-domain repertoire excluding Pkinases in the 13 plant species investigated is illustrated as Figure 1B with a color code that illuminates the six different R-domains (*i.e.* for a total of 7743 LRR, 3453 NBS, 963 TIR, 171 LysM, 1042 WRKY and 807 RG). Regarding the six different R-domains investigated, LRR and NBS are more abundant in the investigated plant genomes (Figure 1B). LRR and NBS consist in, on average, more than 50% and 20% of detected R-genes in both eudicots and monocots respectively. Few WRKY domains were detected in plants (~3.98% and 3.33% in monocot and eudicot species respectively). However, the number of TIR domains appeared much abundant in eudicots than the monocot species (Additional file 1: Table S2) as previously reported [27-31], which may indicated a specific amplification of such domain during rosids paleohistory. The distribution of R-gene families structured into PTI (consisting in LRR-RLK, LRR, LysM, and LysM kinase), ETI (consisting in NBS-LRR), other Pattern Recognition Receptors (PRRs) divided into R-domains combinations (including NBS, TIR, RG, NBS-Pkinase, NBS-WRKY, TIR-NBS, TIR-NBS-Pkinase, TIR-Pkinase, hereafter ‘R-combination’) and genes involved in disease resistance pathway (including WRKYs and protein-kinases, hereafter ‘R-pathway’) is available as Additional file 1: Figure S3. The observed distribution of R-gene families in the investigated species from the more abundant is R-pathway (13189) > PTI (5844) > R-combination (2525) > ETI (2070).

In order to reconstruct the ancestral R-genes repertoire in plants, we used the recently reconstructed ancestral monocot (5 protochromosomes) and eudicot (7 protochromosomes) karyotypes to investigate the R-genes evolutionary dynamic. In Figure 1C, the evolutionary scenario of the modern grass genomes deriving from a n = 5 ancestor is illustrated [66]. Circular distributions illuminated the conservation rate of R-genes families excluding Pkinases (Figure 1C top) and their abundances within the different species (Figure 1C bottom). In the four grasses, the distribution of R-domains appeared very similar, except for RG, more abundant in *Brachypodium* compared to the other grasses investigated (Fisher Exact Test *P-value* = 4.10E-08, 1.46E-09 and 7.38E-08 in comparison to rice, sorghum, and maize; illustrated as red star in Figure 1C bottom). This phenomenon can simply be explained by the differences in RGA annotation and functional characterization efforts in the different species investigated. Finally, WRKY appeared less abundant in rice compared with the other grasses (Fisher Exact Test *P-value* = 3.41E-04, 2.70E-05, 9.47E-07 in comparison to rice *Brachypodium*, sorghum, and maize; illustrated as blue star in Figure 1C bottom). According to the reconstructed five protochromosomes, dating back to ~50-70 mya before the speciation of the four modern species investigated (containing 1622, 732, 845, and 832 R-genes excluding Pkinase domains in rice, *Brachypodium*, sorghum, and maize respectively), we were able to reconstruct a minimal founder (conserved) pool of 465 ancestral R-domains consisting in 361 LRR, 54 NBS, 6 TIR, 11 LySM, 42 WRKY, and 52 RG (Figure 1C top, Additional file 3: Dataset S2). Based on the same strategy, the evolutionary scenario of the eudicots has been used to unravel a minimal founder pool of 150 R-genes (Additional file 1: Figure S4). In order to understand in more details the evolution of the major PTI/ETI families, we have reconstructed their ancestral pools. The results suggests that PTI genes content in the ancestors are significantly higher than observed in each modern species (*P-value* = 6.33E-04, 3.98E-04, 3.37E-04 in grasses ancestor, rice-*Brachypodium* ancestor and sorghum-maize ancestor respectively, Additional file 1: Figure S5), while the ETI genes content is lower in ancestors compared to modern species (*P-value* = 1.12E-04, 5.03E-04, 3.97E-04 in grasses ancestor, rice-*Brachypodium* ancestor and sorghum-maize ancestor respectively). This result may suggest an opposite evolutionary trend between PTI and ETI families that are respectively lost and gained in the course of evolution.

### R-genes plasticity in response to duplication events

**Figure 2:**
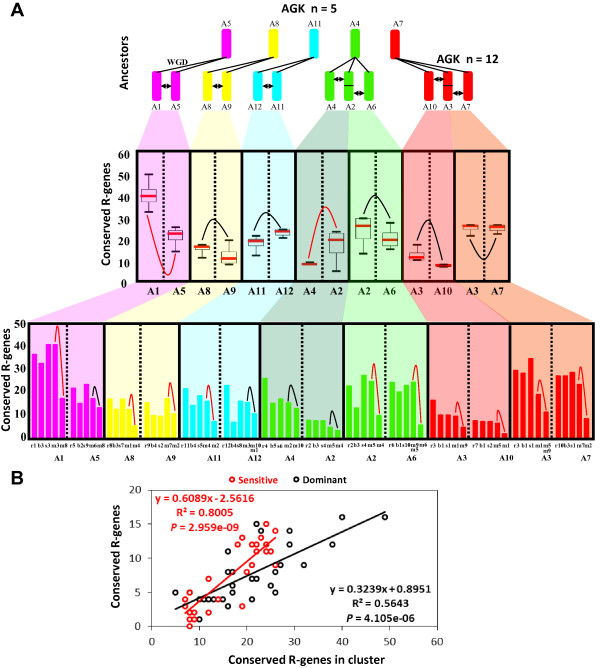
**R-genes conservation between duplicated blocks in grasses. (A)** Illustration at the top of the grass ancestral genomes with n = 5 (A5, A7, A11, A8, A4) paleoduplicated into n = 12 (A1 to A12) defining 7 shared duplicated blocks (black arrows). The number of conserved R-genes (y-axis) between duplicated chromosomes (x-axis) in modern grasses is illustrated as box plots ( illustrates non-significant differences;  illustrates significant differences based on permutation test with *P-value* < 0.05, see Methods section). Distribution pattern of conserved R-genes in modern chromosome pairs is shown in rice, *Brachypodium*, sorghum and maize. **(B)** Illustration of the correlation between the number of observed R-genes in cluster (x-axis) and the total number of conserved R-genes (y-axis) characterized in the sensitive (red dot and curve) and dominant (black dot and curve) chromosomal blocks.

We wanted to investigate the impact of polyploidy (or whole genome duplication, hereafter WGD) in shaping the modern R-genes repertoire deriving from a founder pool of 465 (dating back from ~100 mya) and 150 (dating back from ~250 mya) R-genes for respectively the grasses and rosids. While massive duplicated gene deletion in the course of evolution following WGD has been reported in the literature [68,69], then leading to orthologous dominant (*i.e.* retention of ancestral genes) and sensitive (*i.e.* deletion of ancestral genes) blocks, the particular evolutionary fate of R-genes in response of such diploidization phenomenon is still not well established. To understand the R-genes family plasticity in response to WGDs, we used monocots as a model system to investigate the retention of R-genes (excluding Pkinases) in duplicated fragment pairs (Figure 2A). It has been shown that protein-coding genes behave differently in response to this diploidization process. Diploidization resistant genes (*i.e.* gene functions retained as duplicates following WGDs) are mainly transcription factors (TFs), transcription regulators (TRs) as well as miRNAs to a less extent, whereas the remaining gene families are considered as diploidization sensitive in returning to a singleton status after WGDs *via* selective gene deletion between dominant and sensitive chromosomal blocks [70,71]. At the whole genome level, we observed that only ~5% (25 out of 465) ancestral R-genes mapped on the grass ancestor (*n* = 12) were co-retained (*i.e.* paralogous genes observed in the ancestral duplicated chromosome pairs), a much lower rate than the one reported for transcription factors (TF) as well as miRNA genes with up to 50% of observed co-retention of ancestral duplicates [68,72]. Therefore, the observed lower co-retention of ancestral paralogous R-genes may suggest that R-genes act as diploidization sensitive genes in returning to a singleton status after WGD. However, for 71.43% (five out of seven) duplicated chromosome pairs, the previous characterized deletion of the diploidization sensitive R-genes does not follows the subgenome dominance hypothesis (except for A1/A5 and A2/A4; highlighted with red connecting lines in Figure 2A bottom) in deriving dominant and sensitive blocks [68,73]. Instead, we observed that R-genes retention after WGD is equally distributed between ancestral chromosome pairs A8/9, A11/A12, A2/A6, A3/A7, and A3/A10 (permutation test with *P-value* < 5%, Figure 2A and Additional file 1: Table S3) in the four grass species. Intriguingly, after the recent WGD in maize, for 64.29% (nine out of fourteen paralogous pairs highlighted with red connecting lines in Figure 2A bottom) of the duplicated chromosomes, R-genes deletion was partitioned between the paralogous pairs (permutation test with *P-value* < 5%, Additional file 1: Table S4 and Additional file 1: Figure S6).

Such observed absence in R-genes deletion partitioning in grasses between ancestral duplicated chromosomes may be due to R-gene clusters identified as more abundant in ancestral sensitive chromosomes compared to dominant chromosomal compartment (R^2^ = 0.64 with *P-value* = 4.73E-06 for sensitive chromosomes and R^2^ = 0.34 with *P-value* = 3.70E-03 for dominant chromosomes; Figure 2B and Additional file 1: Table S5). For example, between A2 (reported as dominant) and A4 (reported as sensitive), there is no cluster located in the A2 in contrast to seven genes in three clusters on A4, while between A1 (reported as dominant) and A5 (reported as sensitive) more clusters in the A5 (16 genes in five clusters) do not reverse or reduce the reported dominance of A1 (24 genes in ten clusters). In contrast, R-genes clusters in maize did not affected the observed bias retention of duplicated R-genes between paralogous fragments excluding for A5 (m6 *vs* m8), A12 (m3 *vs* m1/10), A2 (m4 *vs* m5) and A4 (m2 *vs* m10); Figure 2A (bottom), Additional file 1: Table S6. This result may indicate that the random deletion of R-genes after WGD, not following the known subgenome dominance rule for the ancestral tetraploidization, may be a consequence of the high plasticity of such gene family evolving particularly in local tandem duplications (also referenced as clusterization in the next section) that may have compensated the ancestral biased deletion of duplicates in known sensitive subgenomes in the course of evolution. However, for the recent WGD in maize dating back to 5 mya, tandem duplications or clusters can’t offset the dominance/sensitivity effect in such short period of time. Thus, our data led to the hypothesis that R-genes, identified as diploidization sensitive genes, may have followed the subgenome dominance hypothesis that was compensate in the course of history by a reshuffling return flow consisting in local tandem duplications, enriching sensitive genomic compartments in R-genes content.

### R-genes plasticity *via* clusterization and transposition mechanisms

While R-genes have been reported to be clustered in grass chromosomes [34] based on few species or loci investigated, a large-scale investigation of this phenomenon is still lacking in monocots and dicots. The structural definition of a R-gene cluster was considered following a previous study where linked (*i.e.* clustered) R-genes were not interrupted by more than eight non-R-genes [74]. In Additional file 1: Table S7, we reported that there are about 69% and 63% R-genes on average organized in clusters in monocots and eudicots respectively, suggesting that R-genes families expanded by lineage-specific tandem duplications leading to duplicated gene copy variants associated with high sequence similarities. Surprisingly, in poplar for example, we detected only 32% of R-genes organized in clusters using the same strategy, then unraveling possible specific patterns of R-gene clusterization between species. The typology of the R-genes clusters differs between species then reinforcing the concept of a recent clusterization process with most (58% on average) of them consisting in clusters of two locally duplicated R-genes, especially in maize (72%), while the largest and rare cluster, made of six R-genes, was only observed in rice (Additional file 1: Figure S6).

**Figure 3:**
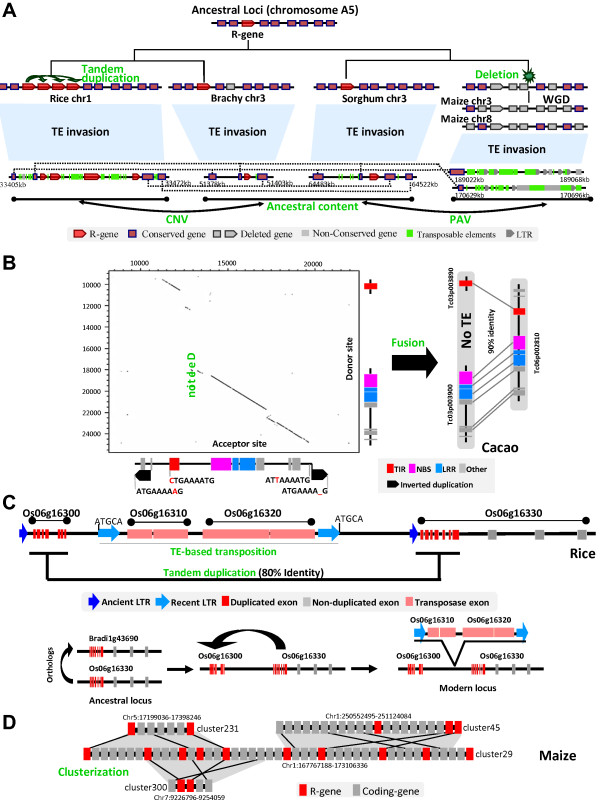
**Different sources of R-genes plasticity in plants. (A)** Evolutionary history of a locus located on the ancestral chromosome A5 showing R-gene conservation as well as CNV and PAV between rice, *Brachypodium*, sorghum and maize. Conserved genes, non-conserved genes, deleted genes and transposable elements are illustrated according to the legend at the bottom. Dotted black lines link orthologous genes between modern loci at the bottom. The ancestral gene content is illustrated at the top. **(B)** Illustration of an example of R-gene fusion in cacao after duplication. The dotplot illustrates the two copy-paste regions flanked by TSD (Target Site Duplication as black arrows) motifs and repeated sequence motifs (ctgaaaatg/attaaaatg). R-genes (TIR, NBS, LRR) classes are illustrated according to the color code at the bottom. **(C)** Illustration of a R-gene (Os06g16330) partially duplicated (Os06g16300) in rice separated by a transposition event (Os06g16310- Os06g16320). The reconstructed evolutionary scenario is illustrated based on a color code illuminating repeat (ancient, recent) and gene (duplicated, non-duplicated, transposase) content. **(D)** Duplicated R-gene cluster plasticity in maize. One central R-gene cluster (chromosome 1) consisting in R-genes (red) and non R-genes (grey) is duplicated on homeoelogous regions (chromosomes 1-5-7) with distinct R-gene contents.

The particular evolution of R-genes *via* clusterization was highly dynamic through lineage-specific rearrangements leading to the observed conservation/erosion of R-genes colinearity between grasses, referenced as Copy Number Variation (CNV) and Presence/Absence Variation (PAV). The Figure 3A illustrates an orthologous R-gene locus in grasses involving a 178 kb rice region on chromosome 1 (containing four R-genes in clusters), a 68 kb region on the *Brachypodium* chromosome 2 (a single R-gene), a 161 kb region of the sorghum chromosome 3 (a single R-gene), and the two orthologous regions in maize on chromosomes 3 (232 kb) and 8 (113 kb) both with no R-gene annotated. This example illustrates the extreme structural variation in R-gene content between orthologous regions. On the two maize paralogous regions no R-gene were identified, while transposable elements (TEs) are found with high concentration (green rectangles as shown in Figure 3A). Their presences suggest that TEs may be involved in the loss of R-genes through illegitimate recombination. The reconstruction of the evolutionary history of this locus illustrates the plasticity of the R-gene family where a single ancestral R-gene is retained in the modern *Brachypodium* and sorghum genomes but evolved into CNV in rice (four copies) and PAV (no R-gene) in maize.

The Figure 3B illustrates the retention of duplicated loci with 90% of sequence similarity in cacao, one locus with one R-gene (Tc06p002810 consisting TIR-NBS-LRR domains), and the duplicated locus harboring two R-genes (*i.e.* Tc03p00890 with a TIR domain and Tc03p00900 with NBS-LRR domains). We located precisely TSD (Target Site Duplication) motifs and long terminal reverse duplication, suggesting Tc06p002810 as the acceptor site (Figure 3B, Dotplot horizontal axis) and the duplicated region with two genes as the donor site (Figure 3B, Dotplot vertical axis). The acceptor site is characterized by a 7 kb fragmental deletion, which is located in the intergenic region of the donor site between Tc03p00890 and Tc03p00900 (with no transposable element or repeat detected in this particular fragment). The deletion of the intergenic fragment between the neighbor genes may have led to the read through of the ORFs leading the two neighbor genes fused into a single one (Tc06p002810) in the course of evolution. These paralogous regions from cacao may then suggest duplication as a major process resulting in domain shuffling (then reducing colinearity between species) between tandem duplicated R-genes.

Such R-gene structural plasticity may also be driven by TEs as we illustrated in the Figure 3C with two tandem duplicated R-genes with about 80% of sequence similarity from the rice genome, *i.e.* Os06g16300 (LRR domain) and Os06g16330 (LRR-Pkinase domains). Using LTR_Finder [75], we identified a 240 bp ancient LTRs flanking the Os06g16300 gene (dark blue arrow) as well as 5 bp TSD motifs associated with 1.1 kb recent LTRs (light blue arrow) flanking two transposase genes, *i.e.* Os06g16310 and Os06g16320. We then proposed an evolutionary scenario for this locus, where Os06g16300 is the ancestral gene (conserved with the modern *Brachypodium* gene, Bradi1g43690) that have been partially (illustrated as red exons) duplicated in tandem as Os06g16330 and finally physically separated by the TE-based transposition of the two transposases (Os06g16310 and Os06g16320). Overall, this example of tandem duplication followed by TE-based transposition events illustrates another source of R-gene plasticity reported in the current analysis, leading to R-gene synteny erosion between closely related species.

Taking into account the previous case examples obtained from cacao and rice genomes and in order to investigate R-gene synteny erosion at the whole genome level, we aligned the non-syntenic R-genes with the total R-genes repertoire. Using the parameters CIP > = 70% and CALP > = 70% [66] to identify the paired non-self matches, and according to the similarity of flanked protein-coding genes between the paired R-genes using E-value < e-10 as a blast threshold, we distinguished segmental from single-gene duplications (also referenced as Small-Scale Duplication *i.e.* SSD) from these gene pairs (detailed in Methods section). In rice, *Brachypodium*, sorghum, and maize, we found 13.04% (153 out of 1173), 9.08% (74 out of 815), 12.35% (115 out of 931), and 35.63% (404 out of 1134) R-genes loci (R-genes located in the same clusters were considered as a single locus) involved in single-gene duplications (Additional file 1: Table S8 and Additional file 4: Dataset S3), which is higher than the 5% to 7% of single-gene duplication frequency reported for the total annotated protein-coding genes in grasses [76]. Among grasses, the single-gene duplication frequency in maize is significantly higher than in rice, *Brachypodium*, and sorghum respectively (*P-value* = 6.32E-24, 2.28E-29, and 1.71E-22 in Fisher’s Exact Test respectively, *cf* Additional file 1: Table S9). In addition, we observed hotspots of single-gene duplications where R-loci showing higher sequence similarity with at least two other non-related R-loci was considered as hotspot (Figure 3D). In maize, 51.73% (209 out of 404) of the single-gene duplications frequency was observed, a much higher rate compared to 23.53% (36 out of 153), 37.84% (28 out of 74), and 31.30% (36 out of 115) in rice, *Brachypodium*, and sorghum respectively (Additional file 1: Table S8). We can then speculate that the recent WGD in maize, dating back to 5 mya, may have promoted and accelerated R-gene singleton duplication frequency compared to the other grasses.

Homologous R-genes sequences within clusters generated by tandem duplications provided the structural template to form novel R-gene informs though domain recombinations. We characterized all the different R-domains in modern clusters and observed a specific domain affinity for clusterization (Additional file 1: Figure S7A and Additional file 1: Table S2). NBS-LRR and LRR-Pkinase combinations are observed as representing the majority of domain combinations in clusters (on average 31.78% and 42.63% out of the total R-domains in clusters for NBS-LRR, 51.08% and 46.47% for LRR-Pkinase domains, respectively in monocots and eudicots), compared to rare observed combinations in clusters for LRR-NBS-PKinase-WRKY or LRR-TIR-WRKY (Additional file 1: Table S2). More interestingly, we observed a preference or affinity in domain combinations where more than 90% of them included LRR (Additional file 1: Figure S7B), with a preferential observed R-domain association with Pkinase (59% and 52% in monocots and eudicots respectively; Additional file 1: Figure S7C) and NBS (41% and 46% in monocots and eudicots respectively; Additional file 1: Figure S7C) domains. Therefore, our data confirm and largely refine previous conclusions suggesting LRR as a ‘glue’ for domain association leading to new combinations of R-gene domains observed in modern species, one major source of R-gene plasticity. This dynamic recombination of R-domains within clusters, especially enriching NBS-LRR associations, may promote the development a novel source of disease resistance in the investigated species.

### R-gene plasticity mediated by miRNA/R-gene interactome

MiRNAs, as a versatile class of post-transcriptional gene regulator, are reported to be involved in a large variety of cellular processes, including development and defense responses in plants [77-79]. Small RNA cloning and high-throughput sequencing from plants infected by pathogens have shown that many microRNAs [80-82] and siRNAs [83] may be involved in biotic defense responses through up or down regulation of targeted gene expression. We wanted then to investigate whether the miRNA/R-gene interactome had an impact on the R-genes evolutionary plasticity as the role of miRNAs in plant immunity system has been largely reported in the literature [80-82]. To unveil if the plant paleoevolution has affected or even shaped the R-gene/miRNA interactome, we investigated miRNAs potentially targeting R-genes in the four monocots investigated (rice, *Brachypodium*, sorghum, and maize) as well as in nine eudicots (grape, *Arabidopsis*, strawberry, cacao, papaya, poplar, soybean, apple, and lotus).

**Figure 4:**
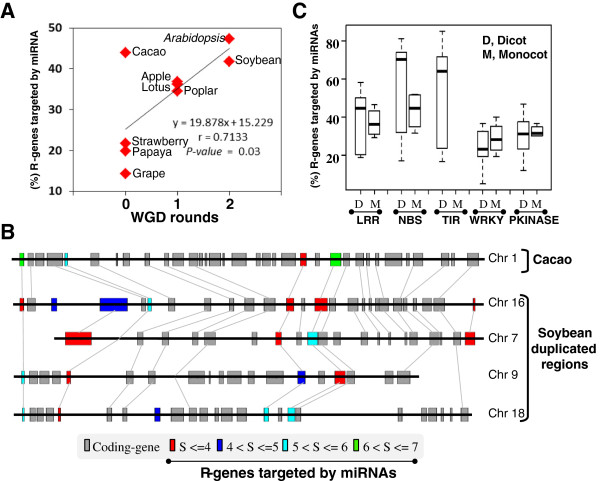
**R-genes/miRNAs interactome in plants. (A)** Illustration of the percentage of R-genes targeted by miRNA in dicots species classified according the number of experienced WGDs (x-axis). The regression curve, correlation and associated *P-value* are mentioned. **(B)** Illustration of a micro-synteny locus between cacao (one region) and soybean (four duplicated regions) harboring R-genes targeted by miRNA (according to the number of sequence mismatches between miRNA and R-genes from 4 to 7 identified as miRNA target score and highlighted with a color code at the bottom). Grey bars represent non R-genes. **(C)** Illustration of the percentage of R-genes targeted by miRNA (y-axis) in dicots **(D)** and monocots (M) species classified according to the investigated R-domains (LRR, NBS, TIR, WRKY, Pkinase; x-axis).

We considered resistance genes as *in silico* targets of miRNAs based on sequence mismatch scores using Targetfinder algorithm [84] (detailed in Methods section). On average, we characterized 33.31% and 35.60% R-genes predicted as *in silico* targets of miRNA in monocots and eudicots respectively, significantly higher than for non R-genes with 11.48% and 13.06% respectively (*P-value* = 9.013e-05 in paired student t-test, Additional file 1: Table S10). No highly significant differences where observed between non-conserved and conserved R-genes targeted *in silico* by miRNAs in monocot (Table 2). In eudicots, these differences (*P-value* = 3.48E-02 between conserved and non-conserved miRNA-targeted resistance genes) are likely to be associated with the numerous rounds of WGD. We observed a correlation (r = 0.7133 with *P-value* = 0.03) between the number of WGD rounds and the number of *in silico* miRNA/R-gene interactions that took place in the plant paleohistory (Figure 4A). This observation may suggest that successive WGDs may have increased or putatively shaped the R-gene/miRNA *in silico* interactome. After recent WGDs, for example, in soybean, ~50% of retained R-genes (Figure 4B) are potential targeted by miRNAs with mismatch score of < = 4. One explanation could be that additional species-specific R-genes copies (deriving from lineage-specific WGDs), then leading to R-gene functional redundancy, may be repressed at the expressional level through miRNAs. Such suggested impact of miRNA regulation on duplicated R-genes expression may need to be biologically and functionally validated.

**Table 2:** miRNA repertoire targeting conserved and lineage-specific R-genes in plants

**Lineage-specific WGD rounds**	**Conserved**^**1**^				**Non-conserved**^**2**^		
	**Species**	**Targets**^**3**^	**Total orth**^**4**^	**(%)**^**5**^	**Targets**^**3**^	**Total non-orth**^**4**^	**(%)**^**5**^
**Monocot**							
**0 WGD**	OS	178	521	34.17	771	2116	36.44
**0 WGD**	BD	156	495	31.52	319	1167	27.34
**0 WGD**	SB	158	413	38.26	489	1304	37.50
**1 WGD**	ZM	105	319	32.92	448	1548	28.94
**Eudicot**							
**0 WGD**	VV	29	282	10.28	124	796	15.58
**0 WGD**	TC	77	149	51.68	554	1290	42.95
**0 WGD**	CP	22	101	21.78	116	602	19.27
**0 WGD**	FV	39	108	36.11	275	1344	20.46
**1 WGD**	MD	49	125	39.20	1516	4127	36.73
**1 WGD**	PT	59	122	48.36	389	1175	33.11
**1 WGD**	LJ	23	46	50.00	219	622	35.21
**2 WGD**	AT	51	74	68.92	685	1485	46.13
**2 WGD**	GM	72	148	48.65	1307	3162	41.33

Note: OS, BD, SB, ZM, VV, TC, CP, FV, MD, PT, LJ, AT, and GM represent Rice, *Brachypodium*, Sorghum, Maize, Grape, Cacao, Papaya, Strawberry, Poplar, Lotus, *Arabidopsis*, and Soybean, respectively; ^1^Conserved R-genes are associated with orthologous genes with other monocots and eudicots species; ^2^Non-conserved R-genes do not have orthologous genes with other species either monocots and eudicots; ^3^Number of R-genes targeted by miRNAs; ^4^Number of total orthologous genes; ^5^Percentage of R-genes targeted by miRNAs.

Finally, we investigated the R-domains/miRNA affinity and observed that NBS/TIR > LRR > WRKY/PKinase domains are preferentially targeted by miRNAs (*P-value* = 2.17E-03 and 8.45E-03, 1.21E-03, and 3.97E-03 with paired student t-test for NBS *vs* LRR, TIR *vs* LRR, LRR *vs* WRKY, LRR *vs* Pkinase, respectively; Figure 4C & Additional file 1: Table S11). We also observed that 67% and 63% of R-genes clusters are targeted by miRNA in contrast to 33% and 37% of singleton R-genes, respectively in eudicots and monocots (Additional file 1: Figure S8 and Additional file 1: Table S12). Overall, the interaction affinity between species-specific R-genes (in clusters and in majority involving NBS/TIR domains) and miRNAs after WGD can be considered as a major source of R-gene family plasticity in plants, as part of a possible functional diploidization of structurally retained duplicated R-genes.

## Discussion

### Diploidization following duplication as a major source of R-genes structural plasticity

Most of the investigated rosids (grape, *Arabidopsis*, soybean, poplar, and cacao) species experienced up to three WGD events, whereas the investigated grasses (rice, maize, sorghum, and *Brachypodium*) went through one shared ancestral WGD during their evolution, except for maize which experienced a recent extra-WGD 5 mya [66]. Biased erosion of duplicated gene redundancy between sister blocks has been characterized recently in plants defining dominant and sensitive blocks [68,69]. In our current analysis, the identification of 23641 R-genes sequences in angiosperms established a higher R-genes conservation in grasses (on average 23.8%) compared to rosids (on average 6.6%) suggesting that successive rounds of WGDs act as a decay into R-genes conservation, as a primer source of R-gene plasticity. The evolutionary investigation of the characterized R-genes repertoire allowed the reconstruction of minimal ancestral pool of 465 and 150 founder R-genes respectively for the grasses and rosids.

Tandem duplication or clusterization played an important role in R-genes plasticity leading to structural variations such as CNV/PAV between species, which are thought to contribute to the reported tremendous R-genes diversity [85]. Special expansion of tandem duplications especially in sensitive chromosomes, as a rapid counterbalance flow of the duplicates deletion phenomenon, may have compensate R-genes loss in such chromosomal fragments as an expected consequence of the known diploidization process. R-genes clusters may have been shaped by classically proposed shuffling mechanisms such as replication slippage, segmental duplication *via* homologous/non-homologous unequal crossover, transposition *via* ectopic recombination/TE capture [86]. Such clusters may have been shaped by domain shuffling events [87], domain breakage and fusion so that R-domain combinations such as LRR-NBS-PK-TIR and LRR-NBS-PK-WRKY might be the result of local shuffling and recombination events. Altogether, R-gene clusterization, a center source of plasticity, triggered a serial of reshuffling events to make rapid copy variation leading to PAVs and CNVs between species, as a putative source for R-gene structural diversity. In the current analysis we observed up to 60% of R-genes organized in clusters in grasses and rosids. Genic and intergenic sequence repeats within R-clusters generated by duplication, transpositions and insertions provide a structural template that allows mis-pairing during recombination giving rise to unequal crossovers and interlocus gene conversions/rearrangements. The resulting R-domain combinations appeared not random with LRR as a ‘glue’ for domain association leading to new resistance gene isoforms in modern plant species.

### MicroRNA/R-gene interactome as a major source of R-genes functional diploidization

Recently, new evidences have been proposed regarding miRNAs regulating NBS-LRR in plants such as miR2109/miR2118/miR1507 in Medicago, miR482/miR2118 in tomato, and miR6019/miR6020 in tobacco guiding the cleavage of transcript of NBS-LRRs, and then triggering the secondary phased of siRNA production by RNA-dependent RNA polymerase [88-90]. Thus, MiRNAs may be involved in defense immunity in regulating R-genes expression level. Our *in silico* analysis suggests that miRNA may target preferentially duplicated R-genes either deriving from WGDs and more interestingly from local tandem duplications (*i.e.* clusters). This observation may suggest that the presence of redundant duplicated R-genes copies, when retained after diploidization, may require modification or specialization in expression/regulation through possibly miRNA interaction. Moreover, a specific R-domain affinity was observed for miRNA *in silico* interaction toward LRR/NBS/TIR, which may indicate to some extant a domain preference for R-gene/miRNA interaction. Overall, our data may suggest miRNAs as a dosage regulator playing a possible role in R-genes functional redundancy erosion following large or local duplication events. The preferential post-transcriptional regulation of duplicated R-genes by miRNA can be proposed as part of a functional diploidization process in response to duplications to maintain a perfect dosage balance regarding the product of R-genes duplicates.

### Putative model of R-genes paleohistory in plants

**Figure 5:**
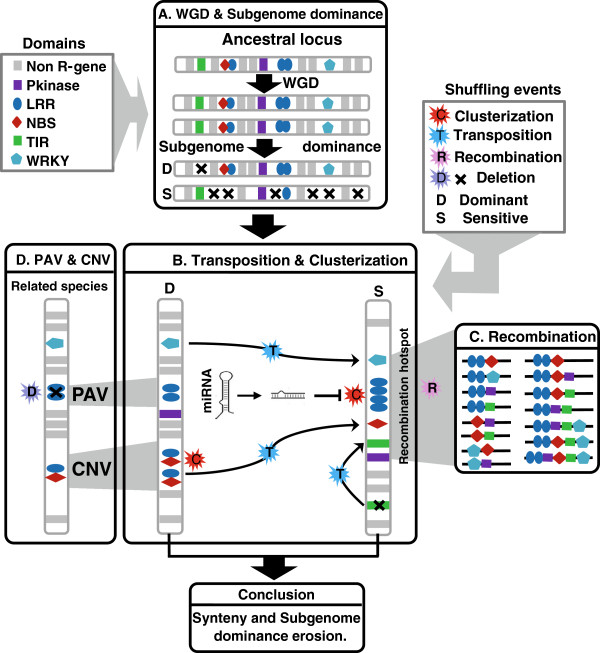
**Evolutionary model of R-genes in plant genomes.** Major conclusions from the current study are schematically illustrated in four panels highlighting **(A)** biased deletion of R-genes between duplicated blocks (D for dominant and S for sensitive) after whole genome duplication; **(B)** R-genes shuffling *via* transposition as well as clusterization involving R-gene/miRNA interactions; **(C)** R-genes domains rearrangement in clusters considered as recombination hotspots; **(D)** Presence/Absence Variation (PAV) and Copy Number Variation (CNV) between related species. R-gene classes are illustrated according to the color code at the top left. Shuffling events are illustrated through the color code at the top right.

Based on our analysis and previous studies, we proposed an hypothetical evolutionary model in Figure 5 illustrating the conclusion regarding the reported R-gene conservation/diversity, polyploidization, domain reshuffling as well as microRNA/R-gene interactome. If duplicated R-genes are deleted between paralogous fragments after whole genome duplications following the general subgenome dominance hypothesis [68,69], they can be then consider as dosage-sensitive genes in returning to singleton status after WGDs and then are biased distributed on the modern pairs of duplicated chromosomes (Figure 5A). However, during evolution, if the deletion of R-genes in sensitive chromosome is compensated by high frequency of tandem duplication (clusterization) and/or transposition events, an equivalent number of R-genes between dominant and sensitive fragments is observed in modern plant genomes (Figure 5B). Despite the previous structural R-genes plasticity scenario, R-genes functional redundancy within clusters or in general between duplicated loci may be counterbalanced through microRNAs regulation with a specific affinity for LRR/NBS/TIR domains. Therefore, R-genes colinearity as well as subgenome dominance following WGDs has been eroded in the course of evolution. Finally R-genes clusterization may be considered as R-domain recombination hotspots, potential source of new domain combinations then possibly facilitating the neo-formation or neo-functionalization of R-genes isoforms (Figure 5C). Overall, when comparing modern plant species for their R-genes content, copy number variation (CNV), or Present/Absent variation (PAV) are generally observed (Figure 5D).

## Conclusions

We reconstructed the R-genes paleohistory in plant unraveling duplications (either whole genome, small-scale clusters or single-gene based) as the major source of structural (CNV, PAV, domain recombination) or even potentially functional (enhanced miRNA regulation for R-gene clusters and specific R-domains) plasticity that may have promote the development a novel source of disease resistance in the course of the evolution of the different investigated species. The conserved role of similar R-gene families (especially TIR and NBS-LRR) in both plant and animal defense systems suggest a common and ancestral origin. The current reconstruction of the ancestral gene pool in angiosperm opens the perspective to determine the origin of innate immunity mechanism in eukaryotes.

## Methods

### R-gene identification and mapping

R-genes were selected from monocots *Oryza sativa, Sorghum bicolor, Zea mays, Brachypodium distachyon,* and eudicots *Arabidopsis thaliana, Populus trichocarpa, Carica papaya, Glycine max, Lotus japonicas, Fragaria vesca,* and *Theobroma cacao*, on the basis of functional annotations available on Phytozome (http://www.phytozome.net/), Plant GDB (http://www.plantgdb.org/). *Vitis vinifera* and *Malus x domestica* R-genes annotation were retrieved from [59] and [60] supplementary data. The R-genes identification methods is illustrated in Additional file 1: Figure S1A. *PFAM domain identification ***–** Putative R-genes were investigated using profile Hidden Markov Model. Several PFAM profiles [64] were used to extract putative R-gene proteins within the 13 genomes investigated: LRR: pf00560, pf07723, pf07725, pf12799, pf01463, pf08263; NB-ARC: pf00931; TIR: pf01582; LysM: pf01476; Pkinase: pf00069; WRKY: pf03106. PFAM profiles were identified within genomes using the hmmsearch algorithm (e-value cut: 1e-10) from HMMER3 (http://hmmer.janelia.org/; [91]). *PRGdb* – R-genes sequences from the Plant Resistance Gene database were downloaded (http://prgdb.cbm.fvg.it/index.php, [65]). Annotation, PFAM and PRGdb –based R-genes were aligned against Soybean (46194 protein sequences), Cacao (27814 protein sequences), Strawberry (34809 protein sequences), Lotus (15470 protein sequences), Papaya (19205 protein sequences), Poplar (30260 protein sequences), Apple (58979 protein sequences), *Brachypodium*, (32255 protein sequences) Sorghum (36338 protein sequences)*,* and Maize (53764 protein sequences) genome data using BLASTP (PFAM and Annotation R-gene sequences) and BLASTX (PRGdb sequences). BLAST results were parsed using CIP (Cumulative Identity Percentage) and CALP (Cumulative Alignment Length Percentage) parameters (70% as minimum threshold) delivering a non-redundant list of R-genes for each species [44].

### Orthologs/Paralogs identification and synteny relationships

Orthologous and paralogous R-genes were identified aligning Rice RefBank against *Brachypodium*, Sorghum and Maize using BLASTALL. BLASTP results are parsed with CIP and CALP parameters set to 60% and 70% as minimum threshold for ortholog identification and 60% and 70% as minimum threshold for paralog identification as described in [44]. Ancestral relationship between monocot species were represented as concentric circles with the visualization tool Circos [92]. Relationship between rosids species were investigated with the same protocol. Grape RefBank was aligned against Soybean, Cacao, Strawberry, Lotus, Papaya, Poplar and Apple genome data. BLAST results were parsed with the same parameters described previously. The synteny relations at the chromosome levels were considered using public synteny data available for both Monocotyledones and Eudicotyledones [66].

R-genes not located in the syntenic region were BLAST aligned against the total R-genes content. The gene pairs excluding self matches (CIP > = 70%, CIAP > = 70%) were considered as single-gene duplication and used to the further analysis. Then we selected 40 flanking genes windows surrounding R-genes pairs. If flanking pairs with E-value < = e-10 are observed these paired R-genes were then considered as part of a segmental duplication, otherwise, as single-gene duplication.

### Permutation-test for R-genes partitioning between duplicated blocks

In the absence of any biased retention/deletion of ancestral R-genes content (N) after WGD (null hypothesis), the post-duplication R-genes content is 2 N = N + N. After million years of evolution and associated shuffling, n1 and n2 R-genes are observed in modern duplicated chromosomes. We simulated the random deletion of R-genes 1000 times (number of deleted R-gene is equal with (N - n1) and (N - n2) in the two duplicated chromosomes respectively), and derived two sample datasets (Sample1: *X*_*1*_, *X*_*2*_, *X*_*3*_……*X*_*1000*_; Sample: *Y*_*1*_, *Y*_*2*_, *Y*_*3*_……*Y*_*1000*_) corresponding to the random deletion of R-genes between blocks. We performed Z-tests (see formula below) to test significant differences in the distribution of retained R-genes between the two duplicated chromosome pairs (null hypothesis rejected).

$$\begin{array}{l} Z=\frac{X-u}{\frac{\sigma }{\sqrt{n}}}\left(u=\frac{\sum_{i=0}^{1000}\left(\mathrm{Xi}-\mathrm{Yi}\right)}{n}\right)\;;\\ X=n1–n2;n=1000;\;\sigma^{2},\mathrm{variance}) \end{array}$$

### MiRNA identification associated with R-genes as targets

Mature miRNAs dataset from miRBase (http://www.mirbase.org/; Release 18) was used to predict R-genes as targets in the investigated plant genomes Targetfinder algorithm (http://carringtonlab.org/resources/targetfinder/) with score < 4 [84]. To reduce the false positive, secondary structures of the identified mature miRNA was validated using MiReNA software [93]. R-genes targeted by miRNA with validated secondary structure and with a mismatch score < 4, are considered as *in silico* targets of miRNA. The detailed pipeline is illustrated in Additional file 1: Figure S1B.

## Competing interests

The authors (RZ, FM, CP, TL and JS) declare they have no competing interests as part of the same research unit (INRA/UBP UMR 1095 GDEC ‘Génétique, Diversité et Ecophysiologie des Céréales’, 5 chemin de Beaulieu, 63100 Clermont-Ferrand, France) when the study performed.

## Authors’ contributions

RZ, FM, and CP performed the analyses. RZ, TL and JS wrote the manuscript. All authors read and approved the final manuscripts.

## Supplementary Material

Additional file 1: Table S1 R-genes conservation in plants. **Table S2.** R-genes domains/family diversity in plants. **Table S3.** Number of R-genes in the ancestral duplicated chromosomes in grasses. **Table S4.** Number of R-genes in the recent duplicated maize chromosomes. **Table S5.** Number of R-genes clusters in ancient duplicated grass chromosomes. **Table S6.** Number of R-genes clusters in recent duplicated maize chromosomes. **Table S7.** R-genes clusters distribution in plants. **Table S8.** R-genes duplication frequency in maize. **Table S9.** R-genes duplication frequency in maize compared to other grasses. **Table S10.** R-genes targeted by miRNAs in plants. **Table S11.** R-domains targeted by miRNAs in eudicots. **Table S12.** R-genes cluster loci targeted by miRNAs in plants. **Figure S1.** R-genes and miRNA detection pipelines. **Figure S2.** R-domains distribution in plant genomes. **Figure S3.** R-genes family distribution in plant genomes. **Figure S4.** R-genes paleohistorical evolution in eudicots. **Figure S5.** Evolutionary scenario of R-genes families in monocots. **Figure S6.** R-genes distribution and content in clusters. **Figure S7.** R-domains combination in clusters. **Figure S8.** R-gene clusters targeted by miRNAs in plants. Click here for file

Additional file 2: Dataset S1 R-gene repertoire and associated miRNAs in plants. The table provides the catalog of R-genes characterized in grasses including rice (OS), *Brachypodium* (BD), sorghum (SB), and maize (ZM), and rosids including grape (VV), *Arabidopsis* (AT), papaya (CP), Cacao (TC), Soybean (GM), Lotus (LJ), Medicago (MD), Stawberry (FV), and Poplar (PT). Click here for file

Additional file 3: Dataset S2 Ancestral R-gene content in grass ancestor. The table provides the catalog of R-genes characterized in the 5 ancestral chromosomes of grasses with associated R-domains and modern R-genes representatives characterized on rice, *Brachypodium*, sorghum and maize duplicated fragments. Click here for file

Additional file 4: Dataset S3 Duplicated R-genes repertoire in grasses. The table provides the catalog of non-syntenic duplicated R-genes characterized in rice (OS), *Brachypodium* (BD), sorghum (SB), and maize (ZM). Click here for file
