# Supplementary material for: Paleo-evolutionary plasticity of plant disease resistance genes
Source: BMC Genomics. 2014 Mar 12;15:187. doi: 10.1186/1471-2164-15-187 (PMC4234491; doi:10.1186/1471-2164-15-187)

**Table S1: R-genes conservation in plants.** For each species investigated (lines), the number of annotated genes and super-R-genes as well as the number and percentage of orthologs (using as reference genomes rice for monocots and grape for eudicots) are indicated in columns. Fisher's Exact tests for the significant conservation of super-R-genes compared to the total number of annotated genes are provided in the last column (right).

| **Species** | **All genes** | | **Resistance genes** | | ***P-value*** |
| --- | --- | --- | --- | --- | --- |
|  | **Genes #** | **Orth #** | **R-genes #** | **Orth #** |  |
| **Sorghum** | 34008 | 6147 | 1717 | 413 | 2.52E-05 |
| **Maize** | 32540 | 4454 | 1867 | 319 | 5.23E-04 |
| ***Brachypodium*** | 25504 | 8533 | 1662 | 495 | 2.73E-02 |
|  |  |  |  |  |  |
| ***Arabidopsis*** | 33198 | 2389 | 1559 | 74 | 3.54E-04 |
| **Poplus** | 30260 | 4555 | 1297 | 122 | 2.68E-07 |
| **Papaya** | 19205 | 3199 | 703 | 101 | 1.81E-01 |
| **Soybean** | 46164 | 4013 | 3310 | 148 | 2.08E-17 |
| **Apple** | 58979 | 3498 | 4252 | 125 | 4.49E-17 |
| **Lotus** | 15470 | 1720 | 668 | 46 | 1.32E-03 |
| **Strawberry** | 34809 | 3289 | 1452 | 108 | 1.85E-02 |
| **Cacao** | 27814 | 4472 | 1439 | 149 | 1.47E-07 |

**Table S2: R-genes domains/family diversity in plants.** The numbers of different R-domain (lines) combinations are provided for each species (monocots and eudicots) investigated (columns) as well as for the R gene families structured into: ^1^PRR/PTI (LRR-RLK, LRR, LysM, and LysM kinase, in blue), ^2^ETI (NBS-LRR, in red), ^3^other ‘R-combination’ (gene including NBS domain alone without LRR domains and RGA genes, in green), ^4^and ‘R-pathway’ (WRKYs, protein-kinases, in purple).

| **DOMAINS** | **Monocots** | | | | **Eudicots** | | | | | | | | |
| --- | --- | --- | --- | --- | --- | --- | --- | --- | --- | --- | --- | --- | --- |
|  | **Oryza sativa** | ***Brachypodium distachyon*** | **Sorghum bicolor** | **Zea mays** | **Vitis vinifera** | ***Arabidopsis thaliana*** | **Carica papaya** | **Fragaria vesca** | **Glycine max** | **Lotus japonicus** | **Malus x domestica** | **Populus trichocarpa** | **Theobroma cacao** |
| **LRR^1^** | **328** | **173** | **125** | **299** | **151** | **145** | **82** | **246** | **351** | **85** | **732** | **126** | **225** |
| **LRR-Pkinase^1^** | **315** | **163** | **211** | **211** | **116** | **156** | **91** | **145** | **381** | **62** | **331** | **190** | **174** |
| **LRR-Pkinase-Ser/Thr Kinase^1^** | **9** |  | **3** |  |  | **3** |  |  | **8** |  |  |  |  |
| **TIR-LRR-Pkinase^1^** |  |  |  |  |  |  |  | **1** |  |  |  |  |  |
| **TIR-LRR^1^** |  |  |  | **1** |  |  |  | **24** |  | **1** | **4** |  |  |
| **TIR-LRR-WRKY^1^** |  |  |  |  |  |  |  | **3** |  |  |  |  |  |
| **LRR-WRKY^1^** |  |  |  |  |  |  |  |  |  |  |  |  | **2** |
| **LysM^1^** | **14** | **11** | **14** |  | **7** | **9** | **9** | **3** | **31** |  | **9** | **16** | **3** |
| **LysM-Pkinase^1^** | **2** |  | **6** | **2** | **3** | **3** | **2** |  | **18** |  |  | **8** | **1** |
| **NBS-LRR^2^** | **235** | **98** | **176** | **45** | **121** | **53** | **19** | **52** | **215** | **9** | **420** | **76** | **86** |
| **NBS-LRR-Pkinase^2^** |  | **1** |  |  |  |  |  | **1** |  |  | **3** |  |  |
| **TIR-NBS-LRR-Pkinase^2^** |  |  |  |  | **1** |  |  |  |  |  | **2** |  |  |
| **NBS-LRR-Pkinase-WRKY^2^** |  |  | **1** |  |  | **1** |  |  |  |  |  |  |  |
| **TIR-NBS-LRR^2^** | **2** |  |  | **2** | **32** | **78** | **8** | **11** | **142** | **8** | **145** | **21** | **5** |
| **NBS-LRR-WRKY^2^** |  |  |  |  |  |  |  |  |  |  | **1** |  |  |
| **NBS^3^** | **298** | **100** | **105** | **91** | **65** | **19** | **23** | **75** | **50** | **43** | **224** | **23** | **122** |
| **NBS-WRKY^3^** |  |  | **1** |  |  | **1** |  |  |  |  |  |  |  |
| **NBS-Pkinase^3^** | **1** |  | **1** | **1** |  |  |  |  |  |  | **1** |  | **2** |
| **TIR-NBS-Pkinase^3^** |  |  |  |  |  |  |  |  |  |  | **1** |  |  |
| **TIR-NBS^3^** | **3** |  |  | **1** | **10** | **16** |  | **15** | **4** | **17** | **63** | **2** | **5** |
| **TIR-Pkinase^3^** |  |  |  |  |  |  |  | **3** |  |  | **1** |  |  |
| **RG^3^** | **102** | **104** | **39** | **45** | **91** | **126** |  |  | **146** | **5** | **117** | **32** |  |
| **TIR^3^** | **5** | **2** | **2** | **9** | **7** | **31** | **5** | **91** | **20** | **23** | **124** | **8** | **4** |
| **Pkinase^4^** | **1018** | **930** | **872** | **1041** | **437** | **847** | **422** | **733** | **1603** | **381** | **1952** | **732** | **757** |
| **Pkinase-Ser/Thr Kinase^4^** | **205** |  | **65** |  |  |  |  |  | **162** |  |  |  |  |
| **Pkinase-WRKY^4^** | **1** |  |  |  |  |  |  | **1** |  |  | **1** |  |  |
| **WRKY^4^** | **99** | **80** | **96** | **106** | **37** | **71** | **42** | **48** | **179** | **34** | **121** | **63** | **53** |
| Total | **2637** | **1662** | **1717** | **1854** | **1078** | **1559** | **703** | **1452** | **3310** | **668** | **4252** | **1297** | **1439** |

**Table S3:** **Number of R-genes in the ancestral duplicated chromosomes in grasses.** The table provides the number of R genes and associated P-values (calculated by the permutation test method, see the material and methods) for the n=5/12 paleoancestor (AGK), rice (OS) Brachypodium (BD), sorghum (SB) and maize (ZM) genomes.

|  | **N=5 AGK** | **N=12 AGK** | ***P-value*** | **OS** | ***P-value*** | **BD** | ***P-value*** | **SB** | ***P-value*** | **ZM** | ***P-value*** |
| --- | --- | --- | --- | --- | --- | --- | --- | --- | --- | --- | --- |
| **A1** | 108 | 72 | 8.09E-06 | 38 | 8.85E-03 | 32 | 3.54E-03 | 40 | 8.48E-03 | 49 | 6.85E-04 |
| **A5** |  | 40 |  | 21 |  | 14 |  | 23 |  | 25 |  |
| **A8** | 46 | 25 | 5.38E-01 | 16 | 3.81E-01 | 11 | 6.14E-01 | 17 | 3.70E-02 | 16 | 2.66E-01 |
| **A9** |  | 22 |  | 12 |  | 9 |  | 8 |  | 19 |  |
| **A11** | 70 | 39 | 4.85E-01 | 21 | 1.14E+00 | 12 | 7.55E-02 | 19 | 5.41E-01 | 18 | 1.78E-01 |
| **A12** |  | 43 |  | 22 |  | 5 |  | 16 |  | 23 |  |
| **A2** | 85 | 46 | 4.41E-01 | 22 | 1.31E+00 | 13 | 9.18E-02 | 29 | 2.33E-01 | 29 | 4.13E-01 |
| **A6** |  | 41 |  | 24 |  | 20 |  | 22 |  | 24 |  |
| **A2** | 47 | 13 | 4.00E-06 | 8 | 1.78E-05 | 8 | 4.57E-02 | 8 | 1.98E-02 | 9 | 4.06E-03 |
| **A4** |  | 36 |  | 27 |  | 15 |  | 17 |  | 21 |  |
| **A3** | 32 | 18 | 4.75E-01 | 17 | 5.31E-03 | 10 | 3.38E-01 | 10 | 3.85E-01 | 12 | 2.33E-01 |
| **A10** |  | 15 |  | 8 |  | 7 |  | 7 |  | 8 |  |
| **A3** | 77 | 44 | 2.02E-01 | 26 | 9.78E-01 | 21 | 3.04E-01 | 26 | 4.94E-01 | 25 | 4.25E-01 |
| **A7** |  | 36 |  | 26 |  | 24 |  | 22 |  | 26 |  |

**Table S4: Number of R-genes in the recent duplicated maize chromosomes.** The table provides the number of R genes and associated *P-values* (calculated by the permutation test method, see the material and methods) for the maize duplicated regions deriving from the n=12 paleoancestor (AGK).

| **AGK (*n = 12*)**  **Chromosomes** | **Maize**  **Chromosome** | **Maize**  **R genes #** | **AGK (*n = 12*)**  **R-genes #** | **Permutation**  ***P-value*** |
| --- | --- | --- | --- | --- |
| **A1** | m3 | 40 | 49 | 1.34E-05 |
|  | m8 | 18 |  |  |
| **A5** | m6 | 16 | 25 | 2.70E-01 |
|  | m8 | 12 |  |  |
| **A8** | m1 | 14 | 16 | 6.99E-03 |
|  | m4 | 6 |  |  |
| **A9** | m7 | 17 | 19 | 1.10E-02 |
|  | m2 | 10 |  |  |
| **A11** | m4 | 13 | 18 | 5.05E-02 |
|  | m2 | 7 |  |  |
| **A12** | m3 | 16 | 23 | 7.46E-02 |
|  | m10 | 7 |  |  |
|  | m1 | 3 |  |  |
| **A2** | m5 | 22 | 29 | 2.55E-03 |
|  | m4 | 10 |  |  |
| **A6** | m9 | 16 | 24 | 1.61E-07 |
|  | m5 | 6 |  |  |
|  | m6 | 4 |  |  |
| **A2** | m4 | 6 | 9 | 6.25E-01 |
|  | m5 | 5 |  |  |
| **A4** | m2 | 15 | 21 | 3.39E-01 |
|  | m10 | 12 |  |  |
| **A3** | m1 | 9 | 12 | 4.70E-02 |
|  | m9 | 4 |  |  |
| **A10** | m1 | 2 | 8 | 1.21E-02 |
|  | m5 | 7 |  |  |
| **A3** | m1 | 18 | 25 | 2.42E-02 |
|  | m5 | 5 |  |  |
|  | m9 | 5 |  |  |
| **A7** | m2 | 8 | 26 | 1.02E-05 |
|  | m7 | 24 |  |  |

**Table S5: Number of R-genes clusters in ancient duplicated grass chromosomes.** The table provides for n=12 ancestral chromosomes (Chromosomes A1 to A12 in lines) the number of R genes (GN #, number of genes) and clusters (CLS #, number of clusters) observed in the paleoancestor (AGK), rice (OS) *Brachypodium* (BD), sorghum (SB) and maize (ZM) genomes.

| **Chr** | **AGK** | **GN #** | **CLS #** | **OS** | **GN #** | **CLS #** | **BD** | **GN #** | **CLS #** | **SB** | **GN #** | **CLS #** | **ZM** | **GN #** | **CLS #** |
| --- | --- | --- | --- | --- | --- | --- | --- | --- | --- | --- | --- | --- | --- | --- | --- |
| **A1** | 72 | 24 | 10 | 38 | 6 | 3 | 32 | 8 | 4 | 40 | 14 | 7 | 49 | 11 | 4 |
| **A5** | 40 | 16 | 5 | 21 | 4 | 2 | 14 | 6 | 3 | 23 | 11 | 3 | 25 | 8 | 3 |
| **A8** | 25 | 10 | 3 | 16 | 4 | 2 | 11 | 0 | 0 | 17 | 5 | 2 | 16 | 2 | 1 |
| **A9** | 22 | 13 | 5 | 12 | 4 | 2 | 9 | 6 | 3 | 8 | 4 | 2 | 19 | 6 | 3 |
| **A11** | 39 | 12 | 5 | 21 | 2 | 1 | 12 | 4 | 2 | 19 | 6 | 3 | 18 | 7 | 3 |
| **A12** | 43 | 13 | 6 | 22 | 4 | 2 | 5 | 2 | 1 | 16 | 6 | 3 | 23 | 6 | 3 |
| **A2** | 46 | 25 | 10 | 22 | 8 | 3 | 13 | 12 | 6 | 29 | 20 | 8 | 29 | 8 | 4 |
| **A6** | 41 | 12 | 4 | 24 | 4 | 2 | 20 | 5 | 2 | 22 | 9 | 4 | 24 | 8 | 4 |
| **A2** | 13 | 0 | 0 | 8 | 0 | 0 | 8 | 0 | 0 | 8 | 0 | 0 | 9 | 0 | 0 |
| **A4** | 36 | 7 | 3 | 27 | 2 | 1 | 15 | 3 | 1 | 17 | 0 | 0 | 21 | 4 | 2 |
| **A3** | 18 | 6 | 3 | 17 | 4 | 2 | 10 | 2 | 1 | 10 | 4 | 2 | 12 | 2 | 1 |
| **A10** | 15 | 8 | 4 | 8 | 2 | 1 | 7 | 2 | 1 | 7 | 2 | 1 | 8 | 6 | 3 |
| **A3** | 44 | 13 | 5 | 26 | 4 | 2 | 21 | 7 | 3 | 26 | 8 | 4 | 25 | 0 | 0 |
| **A7** | 36 | 14 | 6 | 26 | 2 | 1 | 24 | 10 | 5 | 22 | 6 | 3 | 26 | 8 | 4 |

**Table S6: Number of R-genes clusters in recent duplicated maize chromosomes.** The table provides for n=12 ancestral chromosomes (Chromosomes A1 to A12 in lines) the number of R-genes (GN #, number of genes), R-genes in cluster (GNC #, number of genes in clusters) and clusters (CLS #, number of clusters) observed in maize (ZM) duplicated chromosomes genomes.

| **AGK** | **chromosome** | **GN #** | **GNC #** | **CLS #** |
| --- | --- | --- | --- | --- |
| **A1** | m3 | 40 | 14 | 7 |
|  | m8 | 18 | 3 | 3 |
| **A5** | m6 | 16 | 6 | 3 |
|  | m8 | 12 | 5 | 3 |
| **A8** | m1 | 14 | 3 | 2 |
|  | m4 | 6 | 0 | 0 |
| **A9** | m7 | 17 | 2 | 1 |
|  | m2 | 10 | 2 | 1 |
| **A11** | m4 | 13 | 10 | 5 |
|  | m2 | 7 | 4 | 4 |
| **A12** | m3 | 16 | 9 | 6 |
|  | m10 | 7 | 7 | 6 |
|  | m1 | 3 |  |  |
| **A2** | m5 | 22 | 9 | 6 |
|  | m4 | 10 | 3 | 2 |
| **A6** | m9 | 16 | 15 | 10 |
|  | m5 | 6 |  |  |
|  | m6 | 4 | 0 | 0 |
| **A2** | m4 | 6 | 1 | 1 |
|  | m5 | 5 | 0 | 0 |
| **A4** | m2 | 15 | 3 | 2 |
|  | m10 | 12 | 4 | 3 |
| **A3** | m1 | 9 | 2 | 1 |
|  | m9 | 4 | 2 | 1 |
| **A10** | m1 | 2 | 0 | 0 |
|  | m5 | 7 | 6 | 3 |
| **A3** | m1 | 18 | 5 | 4 |
|  | m5 | 5 | 1 | 1 |
|  | m9 | 5 |  |  |
| **A7** | m2 | 8 | 2 | 2 |
|  | m7 | 24 | 12 | 8 |

**Table S7: R-genes clusters distribution in plants.** OS, BD, SB, ZM, VV, TC, CP, FV, MD, PT, LJ, AT, and GM represent Rice, *Brachypodium*, Sorghum, Maize, Grape, Cacao, Papaya, Strawberry, Poplar, Lotus, *Arabidopsis*, and Soybean, respectively (columns). The number of clusters (Cluster #), the number of super-R-gene in clusters (super-R-gene clusters #), the total number of super-R-genes (Total super-R-genes #) and percentages are provided in lines. R-genes in cluster here also are divided into four categories including PTI, ETI, ‘R-combination’, and ‘R-pathway’.

**Table S8: R-genes duplication frequency in maize. A-**The number of R-genes in singletons and clusters (columns) are indicated in lines for rice (OS), *Brachypodium* (BD), sorghum (SB), maize (ZM) characterized in R-loci, R-loci with Single R-gene duplication (corresponding to single-gene or small-scale duplication SSD), Single R-gene duplication Hotspots (where one R-loci region can be duplicated in other loci at least two times), % R-loci with single R-gene duplication (corresponding to the frequency of single R-gene duplication loci *vs* total R-loci), % Single R-gene duplication hotspots (corresponding to the frequency of hotspot loci *vs* total number single R-gene duplication loci) shown in lines. **B-**The relationship among the singleton R-locus, clustered R-locus, and hotspot R-locus was illustrated in the paleo-duplicated blocks mediated by whole genome duplication and transposed duplication events among the ancestral WGD chromosome pairs (R1 & R5) and non-paralogous chromosomes (R7). Blue, purple, and red rectangles represent genes, R-genes and duplicated genes respectively.

A

|  | **OS (Singletons+Clusters)** | **BD (Singletons+Clusters)** | **SB (Singletons+Clusters)** | **ZM (Singletons+Clusters)** |
| --- | --- | --- | --- | --- |
| **R-loci** | 1173 (687+486) | 815 (449+366) | 931 (557+374) | 1134 (729+405) |
| **R-loci with Single R-gene duplication** | 153 | 74 | 115 | 404 |
| **Single R-gene duplication Hotspots** | 36 | 28 | 36 | 209 |
| **% R-loci with single R-gene duplication** | 13.04 | 9.08 | 12.35 | 35.63 |
| **% Single R-gene duplication hotspots** | 23.53 | 37.84 | 31.3 | 51.73 |

**B**

**Table S9: R-genes duplication frequency in maize compared to other grasses** The number of R-loci (singletons + clusters one cluster considered as one loci independently from the number of R-genes detected); Single R-gene duplication (number of non ortholog duplicated R-loci), % Single R-gene duplication % (Single R-gene duplication percentage) are provided in columns for rice (OS), *Brachypodium* (BD), sorghum (SB), maize (ZM). *P-values* (Fisher’s Exact Test) are provided as last column for the comparison of ZM/OS, ZM/BD and ZM/SB.

| **Species** | **R-loci** | **Single R-gene duplication** | **% Single R-gene duplication** | **Fisher’s Exact Test** |
| --- | --- | --- | --- | --- |
| **ZM** | 1134 | 404 | 35.63 | 6.32E-24 |
| **OS** | 1173 | 153 | 13.04 |
| **ZM** | 1134 | 404 | 35.63 | 2.28E-29 |
| **BD** | 815 | 74 | 9.08 |
| **ZM** | 1134 | 404 | 35.63 | 1.71E-22 |
| **SB** | 931 | 115 | 12.35 |

**Table S10: R-genes targeted by miRNAs in plants.** OS, BD, SB, ZM, VV, TC, CP, FV, MD, PT, LJ, AT, and GM represent Rice, *Brachypodium*, Sorghum, Maize, Grape, Cacao, Papaya, Strawberry, Poplar, Lotus, *Arabidopsis*, and Soybean, respectively; ^1^Number of R-genes can be targeted by miRNAs; ^2^Percentage of resistant genes can be targeted by miRNAs; ^3^randomly selected 600 non-resistant genes ten times. ^4^Paired student t-test was performed on the percentage of genes targeted by miRNAs observed to the total R-gene *vs* non-R-genes.

| **Species** | **Total_R-genes** | | | **NON_R-genes^3^** | | |
| --- | --- | --- | --- | --- | --- | --- |
|  | **targets^1^** | **R-genes** | **%^2^** | **targets^1^** | **R-genes** | **%^2^** |
| **OS** | 949 | 2637 | 35.99 | 676 | 6000 | 11.27 |
| **BD** | 476 | 1662 | 28.64 | 742 | 6000 | 12.37 |
| **SB** | 647 | 1717 | 37.68 | 662 | 6000 | 11.03 |
| **ZM** | 554 | 1867 | 29.67 | 676 | 6000 | 11.27 |
| **Average** | **656.50** | **1970.75** | **33.31** | **689** | **6000** | **11.48** |
| **VV** | 154 | 1078 | 14.29 | 892 | 6000 | 14.87 |
| **TC** | 631 | 1439 | 43.85 | 580 | 6000 | 9.67 |
| **CP** | 139 | 703 | 19.77 | 773 | 6000 | 12.88 |
| **FV** | 314 | 1452 | 21.63 | 1011 | 6000 | 16.85 |
| **MD** | 1565 | 4252 | 36.81 | 163 | 6000 | 2.72 |
| **PT** | 448 | 1297 | 34.54 | 420 | 6000 | 7 |
| **LJ** | 242 | 668 | 36.23 | 210 | 6000 | 3.5 |
| **AT** | 737 | 1559 | 47.27 | 799 | 6000 | 13.32 |
| **GM** | 1380 | 3310 | 41.69 | 2203 | 6000 | 36.72 |
| **Average** | **623.33** | **1750.89** | **35.60**  ^4^*P =* 7.34E-06 | **783.44** | **6000** | **13.06** |

**Table S11: R-domains targeted by miRNAs in eudicots.** Number and percentages of NBS, LRR, TIR, WRKY and Pkinase domains (columns) are provided for the nine eudicots species investigated. T-test (paired student t-test) was performed between the percentage of domain *vs* total R-gene regulated by miRNAs for NBS and LRR, TIR and LRR, LRR and WRKY, and LRR and Pkinase.

| **Species** | **NBS** | **% NBS regulated by miRNA** | **TIR** | **% TIR regulated by miRNA** | **LRR** | **% LRR regulated by miRNA** | **WRKY** | **% WRKY regulated by miRNA** | **Pkinase** | **% Pkinase regulated by miRNA** |
| --- | --- | --- | --- | --- | --- | --- | --- | --- | --- | --- |
| **vv** | 229 | 25.76 | 50 | 24 | 421 | 19.24 | 37 | 5.41 | 557 | 12.03 |
| **at** | 168 | 70.24 | 125 | 64 | 436 | 58.26 | 73 | 36.99 | 1010 | 46.93 |
| **pt** | 122 | 63.11 | 31 | 58.06 | 413 | 44.79 | 63 | 31.75 | 930 | 32.15 |
| **cp** | 50 | 32 | 13 | 23.08 | 200 | 19 | 42 | 14.29 | 515 | 18.64 |
| **gm** | 411 | 73.72 | 166 | 84.94 | 1097 | 54.79 | 179 | 34.64 | 2172 | 37.66 |
| **md** | 860 | 70.58 | 340 | 71.76 | 1638 | 47.01 | 123 | 19.51 | 2292 | 28.1 |
| **lj** | 77 | 74.03 | 49 | 71.43 | 165 | 40.61 | 34 | 23.53 | 443 | 31.38 |
| **fv** | 154 | 17.53 | 148 | 16.89 | 483 | 20.5 | 52 | 23.08 | 884 | 23.53 |
| **tc** | 220 | 81.36 | 14 | 71.43 | 492 | 50.2 | 55 | 32.73 | 934 | 37.58 |
| **Average** | **254.56** | **56.48** | **104.00** | **53.95** | **593.89** | **39.38** | **73.11** | **24.66** | **1081.89** | **29.78** |

**
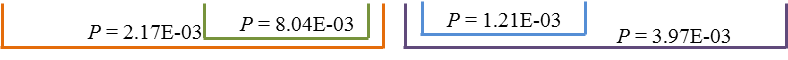
**

**Table S12: R-genes cluster loci targeted by miRNAs in plants. Cluster-loci, if several R-genes (at least two) are clustered distributing on the chromosome, this clustered location as one cluster loci; Cluster miRNA, At least one R-gene in Cluster loci can be targeted by miRNAs; %cluster-miRNA, percentage of cluster-loci regulated by miRNAs in the total cluster loci.**

|  | **Species** | **Cluster-loci #** | **Cluster-miRNA #** | **Cluster-miRNA %** | **Non-cluster #** | **Non-cluster-miRNA #** | **Non-cluster-miRNA %** | ***P-value* (Fisher’s Exact Test)** |
| --- | --- | --- | --- | --- | --- | --- | --- | --- |
| **Eudicots** | ***Arabidopsis*** | 139 | 119 | 85.61 | 398 | 218 | 54.77 | 3.74E-03 |
|  | **Cacao** | 110 | 89 | 80.91 | 229 | 99 | 43.23 | 7.45E-06 |
|  | **Soybean** | 292 | 227 | 77.74 | 650 | 250 | 38.46 | 1.19E-09 |
|  | **Poplar** | 63 | 48 | 76.19 | 424 | 173 | 40.8 | 3.68E-03 |
|  | **Apple** | 460 | 327 | 71.09 | 550 | 240 | 43.64 | 3.85E-06 |
|  | **Lotus** | 47 | 33 | 70.21 | 537 | 177 | 32.96 | 2.98E-03 |
|  | **Papaya** | 48 | 26 | 54.17 | 150 | 22 | 14.67 | 9.75E-05 |
|  | **Stawberry** | 128 | 57 | 44.53 | 340 | 59 | 17.35 | 1.17E-05 |
|  | **Grape** | 102 | 44 | 43.14 | 195 | 22 | 11.28 | 1.80E-06 |
| **Monocots** | **Rice** | 231 | 173 | 74.89 | 436 | 183 | 41.97 | 1.68E-05 |
|  | **Sorghum** | 159 | 109 | 68.55 | 485 | 216 | 44.54 | 4.82E-03 |
|  | **Maize** | 31 | 18 | 58.06 | 769 | 233 | 30.3 | 4.91E-02 |
|  | ***Brachypodium*** | 132 | 69 | 52.27 | 315 | 96 | 30.48 | 4.89E-03 |

**Figure S1: R-genes and miRNA detection pipelines. (A)** Method for R-genes characterization and mapping in plant genomes. **(B)** miRNAs target prediction for R-genes in plants. Calculation of CIP/CALP was performed according to Salse 2012 . miRNA targets were predicted using Targetfinder software (see method section).

**
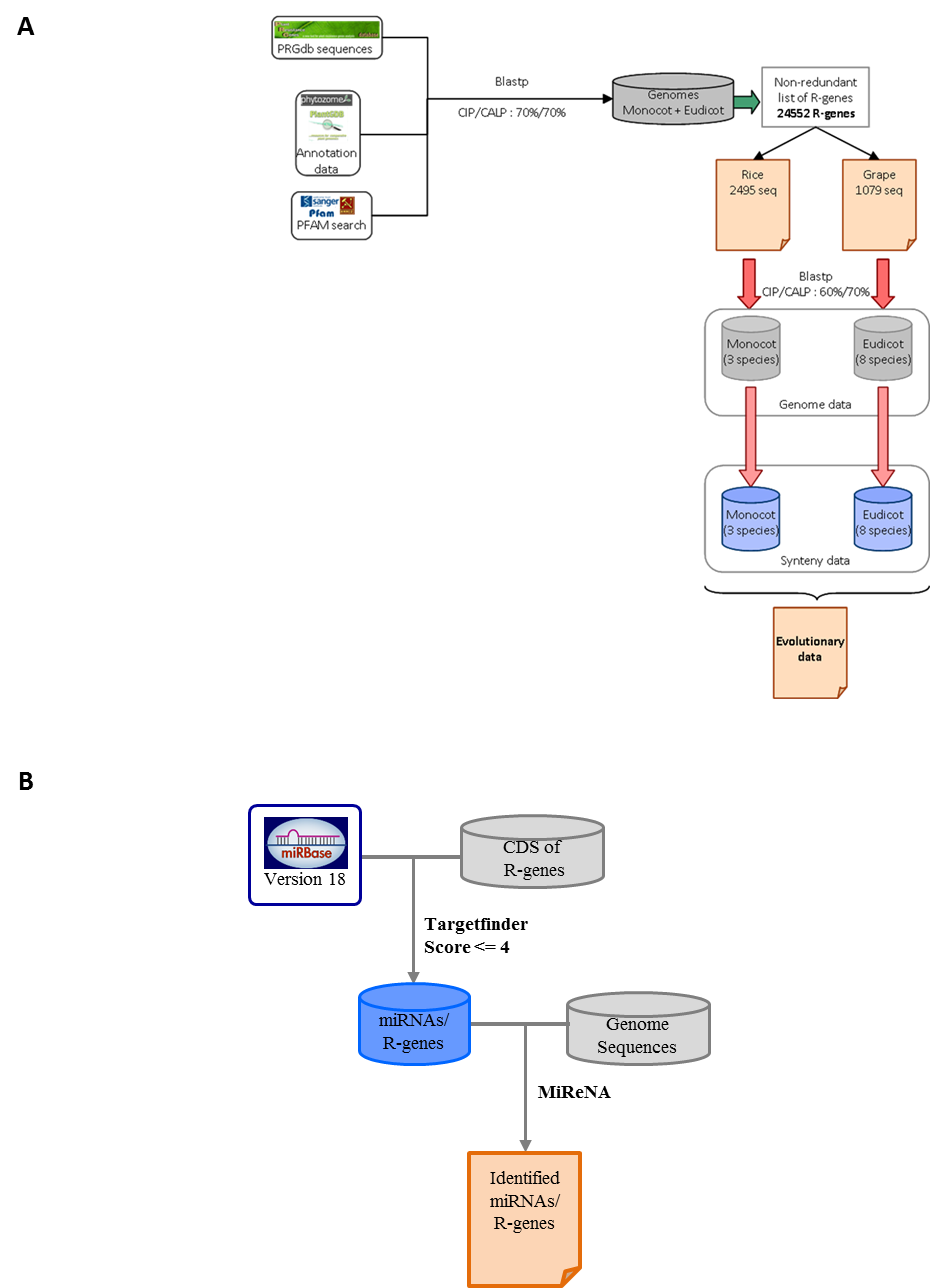
**

**Figure S2: R-domains distribution in plant genomes**. Three results of different methods in detecting R-gens including Annotation (Annot), Plant Resistance Gene database (PRGDB), and PFAM domain (Pfam) are as shown as colored bars. NR, totoal non-redundant R-genes content in integrating the three methods, LRR, NBS, TIR, LysM, RG contents are highlighted with the color code legend at the bottom of the figure.

**
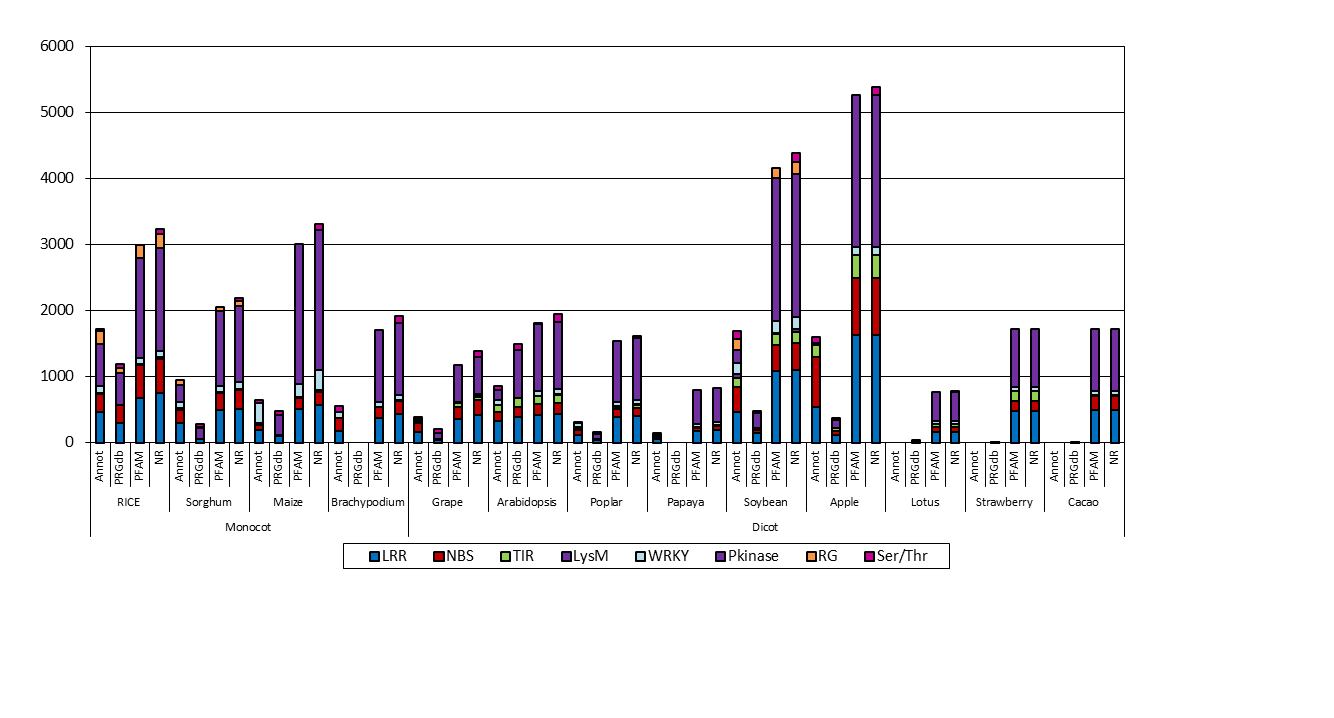
**

**Figure S3:** **R-genes family distribution in plant genomes.** The color code illustrates the R-gene families PTI/ETI/R-combination/R-pathway characterized for the from 13 plant genomes including monocots (rice, *Brachypodium*, sorghum, and maize) and eudicots (*Arabidopsis*, Grape, Cacao, Papaya, Stawberry, Poplar, Lotus, Apple, and Soybean).

**
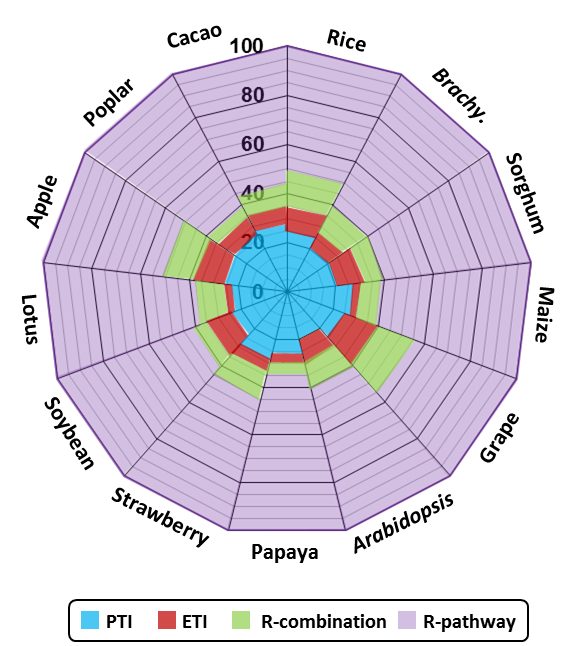
**

**Figure S4: R-genes paleohistorical evolution in eudicots.** The modern genome structures of eudicots are depicted with a seven color code that illuminate their relationships with n = 7 AGK (top) in respectively. The percentages of resistant domains (reference as color code legend at the bottom) are shown with circular distributions for the nine eudicot genomes (bottom) investigated.

***
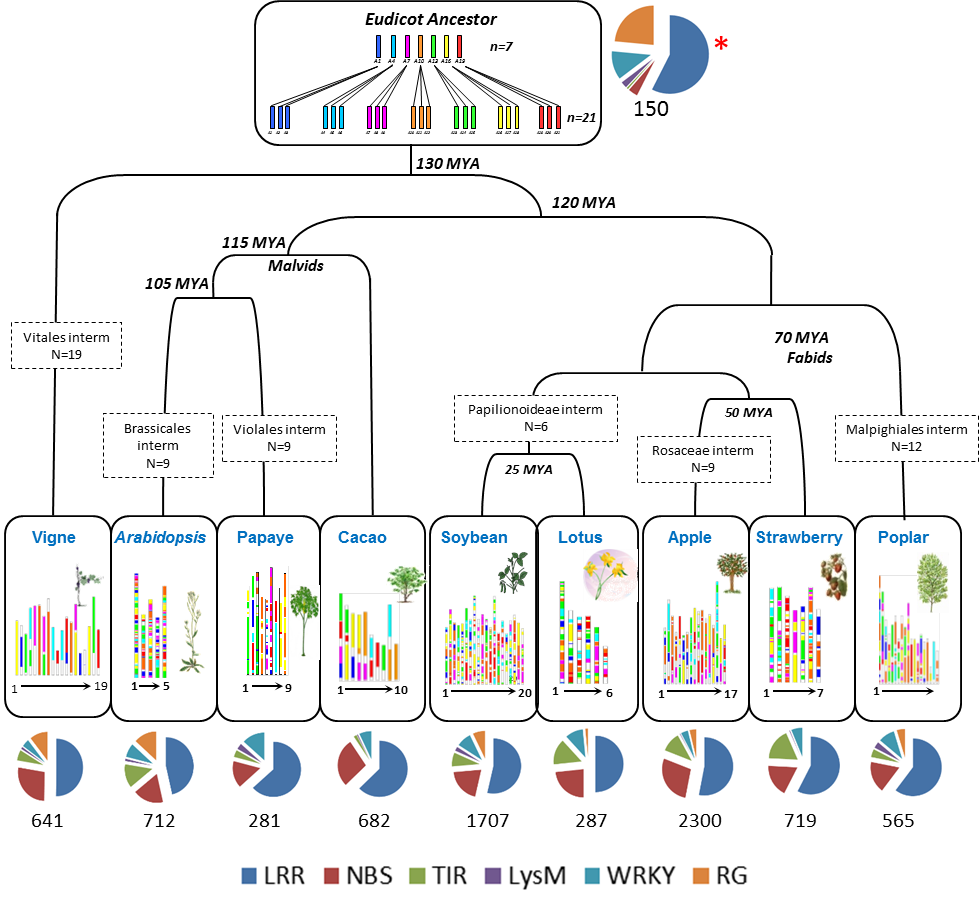
***

**Figure S5: Evolutionary scenario of R-gene families in monocots.** The modern grass genome structures (bottom) are depicted with a five-color code that illuminates their relationship with the n = 5 (A5, A7, A11, A8, A4) and n=12 (A1 to A12) ancestors (top), according to Murat et al. . The characterized R-genes are illustrated as vertical bars on the chromosomes of modern and ancestral genomes. The percentages of R-gene classes (LRR, NBS, TIR, LysM, RG, highlighted with the color code legend at the bottom) are shown with circular distributions for the four monocot genomes (bottom), the rice/*Brachypodium* and sorghum/maize ancestral genome intermediates (center), as well as for the ancestral genomes (top). Statistically enriched and impoverished R-gene families (PTI/ETI/R-combination/R-pathway depicted with a four-color code) are illustrated with red and blue stars on the circular distributions, respectively.

***
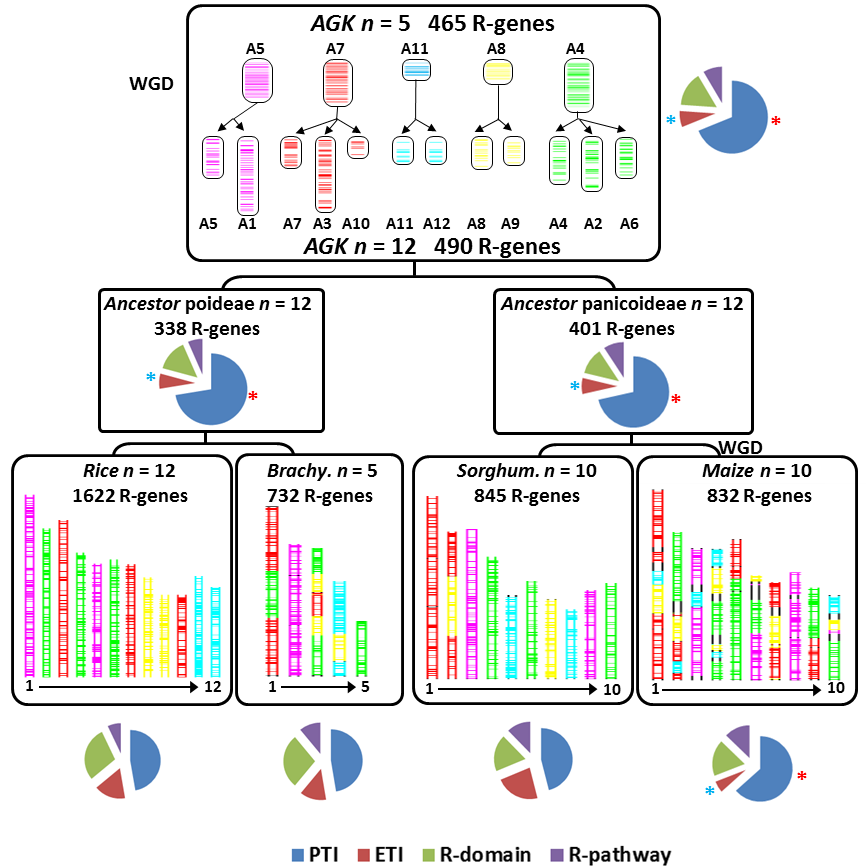
***

**Figure S6: R-genes distribution and content in clusters.** The X-axis represents gene number of R-genes in clusters; the Y-axis represents the percentage of observed clusters with OS for rice BD for *Brachypodium*, SB for sorghum and ZM for maize.

**
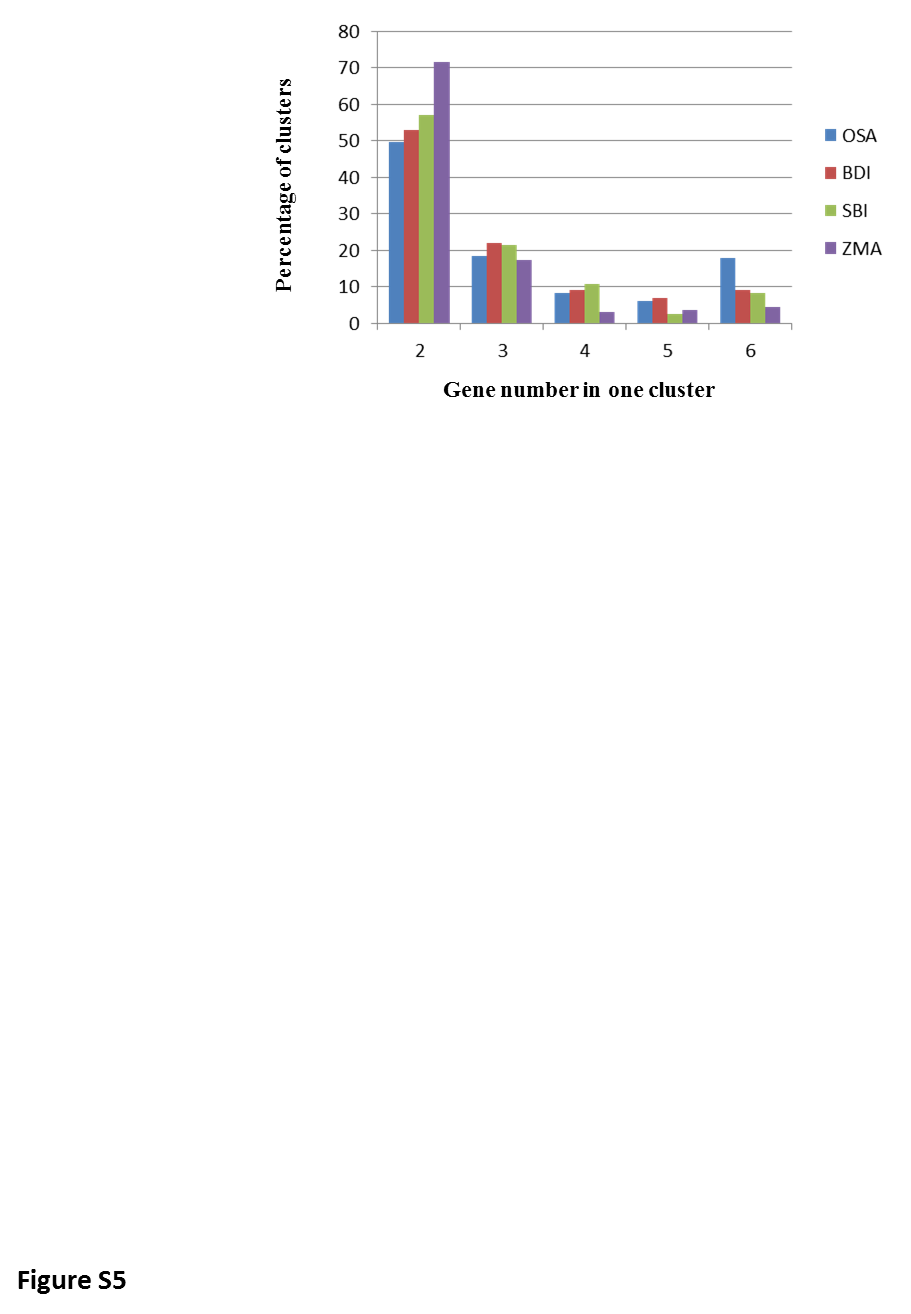
**

**Figure S7:** **R-domains combination in clusters.** **(A)** Illustration of the different domain (color code) combinations observed in the characterized R-gene clusters. **(B)** Domain distribution in the combined R-genes. Y axis represents the percentage of domains and X axis represents the different domains. **(C)** Using LRR domain as a reference, the distribution of the other domains combined with LRR are shown for O, B, S, Z, V, A, C, F, G, L, M, P, T representing rice, *Brachypodium*, Sorghum, Maize, Grape, *Arabidopsis*, Papaya, Strawberry, Soybean, Lotus, Apple, Poplar and Cacao respectively.

**
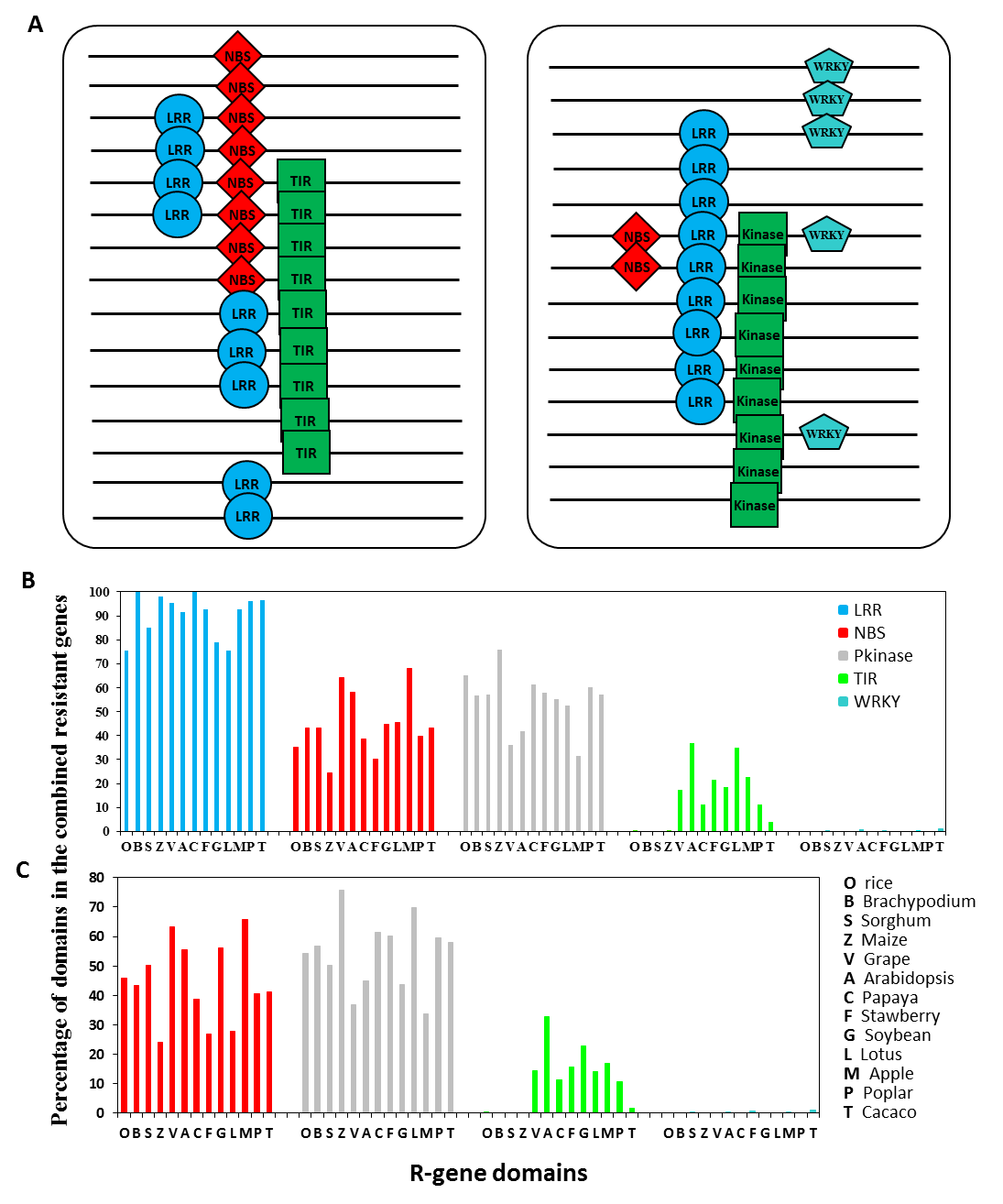
**

**Figure S8:** **R-genes clusters targeted by** **miRNAs in plants.** X-axis represents the investigated species including eudicots (*Arabidopsis*/AT, Cacao/TC, Soybean/GM, Poplar/PT, Apple/MD, Lotus/LJ, Papaya/CP, Strawberry/FV, and grape/VV), and monocots (Rice/OS, Sorghum/SB, Maize/ZM, and *Brachypodium*/BD); Y-axis represents the percentage of R-genes loci regulated by miRNAs (either cluster or non cluster).


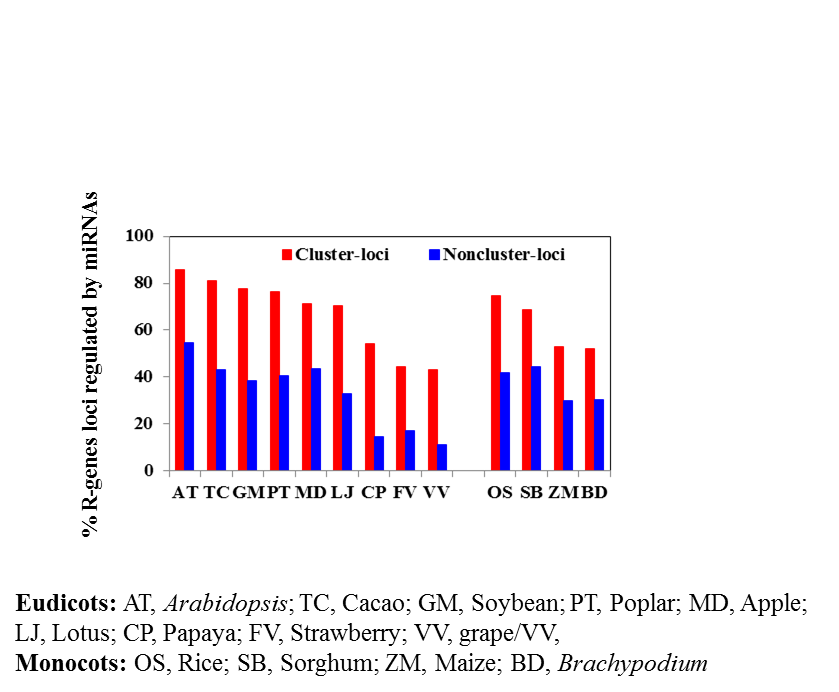

Supplement: Additional file 1: Table S1 — R-genes conservation in plants. Table S2. R-genes domains/family diversity in plants. Table S3. Number of R-genes in the ancestral duplicated chromosomes in grasses. Table S4. Number of R-genes in the recent duplicated maize chromosomes. Table S5. Number of R-genes clusters in ancient duplicated grass chromosomes. Table S6. Number of R-genes clusters in recent duplicated maize chromosomes. Table S7. R-genes clusters distribution in plants. Table S8. R-genes duplication frequency in maize. Table S9. R-genes duplication frequency in maize compared to other grasses. Table S10. R-genes targeted by miRNAs in plants. Table S11. R-domains targeted by miRNAs in eudicots. Table S12. R-genes cluster loci targeted by miRNAs in plants. Figure S1. R-genes and miRNA detection pipelines. Figure S2. R-domains distribution in plant genomes. Figure S3. R-genes family distribution in plant genomes. Figure S4. R-genes paleohistorical evolution in eudicots. Figure S5. Evolutionary scenario of R-genes families in monocots. Figure S6. R-genes distribution and content in clusters. Figure S7. R-domains combination in clusters. Figure S8. R-gene clusters targeted by miRNAs in plants. [file 1471-2164-15-187-S1.doc]
